# What can we learn on ALS pathophysiology from iPSC-derived motor neurons harbouring *TARBDP* mutations: a systematic review

**DOI:** 10.1186/s40035-026-00580-2

**Published:** 2026-09-20

**Authors:** Elena Pasho, Alberto Catanese, Edor Kabashi, Sorana Ciura

**Affiliations:** 1 Laboratory of Translational Research for Neurological Disorders, Imagine Institute, INSERM UMR 1163, Université Paris Cité, 75015 Paris, France; 2 Institute of Neuroanatomy, University Clinic RWTH Aachen, 52074 Aachen, Germany

**Keywords:** ALS, TARDBP, TDP-43, iPSC, Motor neuron, Phenotype, Disease modelling, Drug screening

## Abstract

The degenerating motor neurons of amyotrophic lateral sclerosis (ALS) patients are characterized by the accumulation of cytoplasmic aggregates, specifically enriched in ubiquitinated TDP-43. Expressed mainly in the nucleus and partially in the cytoplasm to execute its role in RNA metabolism, the exact mechanisms that bring TDP-43 to aggregate in disease have yet to be described. Unfolding these processes could bring us closer to effective drug development that is drastically lacking in the ALS research field. Induced pluripotent stem cells (iPSCs) offer a promising platform for studying ALS pathogenesis directly in the relevant human genetic backgrounds of ALS patients. Since 2012, more than 30 published studies have investigated altered cellular and subcellular features in iPSC-derived motor neurons from ALS patients harbouring mutations in the *TARDBP* gene (encoding for TDP-43 protein). However, there are discrepancies in the obtained results, which call into question the relevance of this model for ALS disease modelling and its use for drug development efforts. Thus, there is a need in the field for a clear and detailed layout and summary of all the published data on ALS modelling using *TARDBP* mutant iPSC-derived motor neurons. In this systematic review, we analyse all phenotypic assessments that have been done on iPSC-derived motor neurons derived from *TARDBP* ALS patients on the morphology, functionality and the viability of these cells. We also analyse if this model recapitulates ALS pathology in vitro by comparing studies that looked at TDP-43 and neurofilament aggregation, as well as stress granule dynamics. Importantly, we compare the technical details of all the discussed studies, such as differentiation protocol, age and purity of the used motor neurons and quantification method, in order to discuss how all of these parameters affect the observed phenotypes. Lastly, we also expand our review to all other different cell types that have been differentiated from *TARDBP* iPSCs, highlighting non-cell autonomous mechanisms of TDP-43-ALS pathophysiology. By integrating findings across studies, this review identifies commonalities and discrepancies, discusses methodological advancements and limitations, and highlights potential therapeutic targets revealed through *TARDBP* iPSC-derived models.

**Graphical abstract:**

**Figure:**
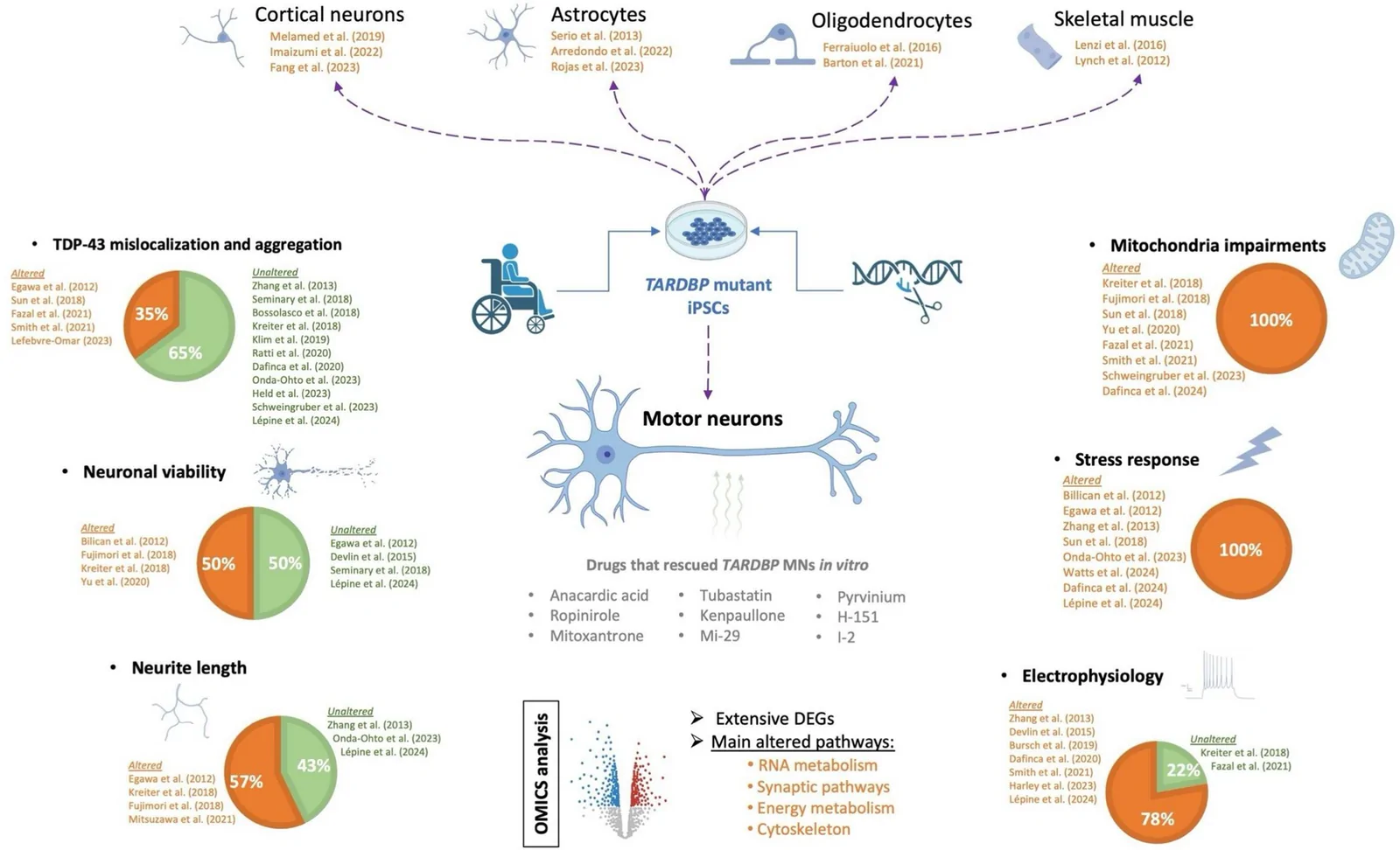

## Background

Amyotrophic lateral sclerosis (ALS) is a neurodegenerative disorder characterized by the progressive degeneration of motor neurons (MNs) in the brain and spinal cord. Even though considered as a rare disease with its yearly incidence of 1.5 and 2 per 100,000 [1], the impacts of ALS on patients, their families and the general society are significant due to its fast progressive and fatal nature. Indeed, MN loss leads in the first place to muscle atrophy and loss of voluntary movement, and within 2 to 5 years to death due to generalized paralysis and respiratory failure [2]. After its first description by French neurologist Jean-Martin Charcot in 1869, scientific research on ALS has been ongoing for more than a century and a half [3]. Despite these extensive efforts, the exact molecular mechanisms underlying ALS remain elusive.

The challenges in understanding ALS pathophysiology are mostly due to the significant heterogeneity observed in both clinical presentations and genetic alterations among ALS patients [4]. ALS is increasingly recognized not as a single disease entity but as a clinical syndrome arising from a combination of genetic and environmental risk factors, encompassing diverse histopathological alterations and clinical manifestations [5]. Over 40 ALS-related genes have been identified, including *SOD1*, *C9orf72*, *TARDBP*, and *FUS*, each contributing differently to disease onset and progression [6]. This genetic diversity leads to distinct molecular mechanisms driving the disease, making it challenging to develop a unified model that accurately represents ALS pathogenesis. ALS has been studied in all the commonly used animal models in research, contributing to most of the understanding of the disease; however, they often fail to fully recapitulate the human disease [5]. Specifically, mouse transgenic models of ALS have been extensively studied [7]; however, they are only able to tackle the understanding of familial forms of ALS, while sporadic forms of ALS with no family history represent 90% of the patients.

A cure for ALS has yet to be achieved and existing treatments such as Riluzole [8] and Edaravone [9] are efficient only in limited subgroups and have been associated with a short survival benefit. More recently, targeted genetic therapies have emerged, including the antisense oligonucleotide tofersen, approved in 2023 for *SOD1*-associated ALS after demonstrating a strong reduction of neurofilament (NF) level, a biomarker of neurodegeneration [10–12]. Other therapeutic strategies are currently under investigation, including masitinib**,** which has shown encouraging results in clinical trials [13], and the mitochondrial- and ER stress-targeting combination therapy AMX0035, which initially showed promise but failed to demonstrate efficacy in a larger phase III trial [14]. Despite these advances, available therapies do not halt disease progression, highlighting the urgent need for improved experimental models to better understand ALS pathophysiology and identify effective therapeutic targets.

Induced pluripotent stem cells (iPSCs) emerged a few years ago as a powerful tool for disease modelling, offering the ability to generate patient-specific pluripotent cells in vitro that could be then differentiated into any cell type. Directly related to this scientific breakthrough, more than a dozen of protocols to differentiate MNs from iPSCs have been published [15], leading to the explosion of studies using iPSCs in the ALS research field with more than 600 publications. Several reviews have discussed the use of iPSC-derived MNs to model ALS [16–18], and more recent articles have summarized findings from iPSC-based ALS studies [19, 20]. However, these were published almost 5 years ago and an updated review is needed as the use of iPSC models has exploded in the last few years. Moreover, most of the reviews discuss ALS modelling with iPSC at a large scale and transcribe results from all ALS causative genes and sporadic cases, which makes the integration of the results overwhelmingly difficult. In this context, we wanted to update the literature review of ALS modelling with iPSC-derived MNs by focusing specifically on one gene: *TARDBP*, which encodes for TAR DNA-binding protein 43 (TDP-43), an RNA- and DNA-binding protein.

Around 2%–5% of familial ALS (fALS) and 1.5% of sporadic cases harbour mutations in the *TARDBP* gene [21]. Normally localized in the nucleus, TDP-43 plays a crucial role in RNA metabolism. However, as a consequence of ALS disease, TDP-43 becomes hyperphosphorylated, cleaved into fragments and aggregated into inclusions in the degenerating MNs of almost all ALS patients (around 97%)[22]. TDP-43 pathology is also a hallmark of many cases of frontotemporal dementia (FTD), supporting the concept that ALS and FTD represent a neurodegenerative disease spectrum [23, 24]. Given the pivotal role of TDP-43 in ALS, understanding how *TARDBP* mutations lead to neurodegeneration will provide great insight into the role of TDP-43 proteinopathy in ALS motor neuron degeneration.

In this context, we selected 36 studies published since 2012 that have focused on characterizing ALS-related phenotypes in iPSC-derived MNs specifically with *TARDBP* mutations. These studies have reported various cellular alterations; however, there is considerable variability in the findings, making it challenging to draw definitive conclusions regarding the reliability of this model in recapitulating ALS pathology. Factors such as differentiation protocols, the purity and age of MNs, and quantification methods could all contribute to these inconsistencies. Thus, a systematic review of existing data is essential for assessing the robustness and reproducibility of this model for ALS research, and more specifically the use for drug screening.

In this systematic review, we aim to provide a detailed summary of all published studies investigating iPSC-derived MNs from *TARDBP*-mutant ALS patients. In order to focus on disease-relevant models, we excluded studies working with overexpression, knock-down or knock-out of TDP-43 in iPSCs. By integrating data from multiple studies, this review aims to highlight key findings, identify commonalities and discrepancies, and provide insights into the strengths and limitations of iPSC-derived MNs as a model for ALS. We also discuss all the other cell types differentiated from *TARDBP* iPSC lines and their cell and non-cell autonomous disease mechanisms. Our goal is to offer a clearer perspective on the potential of iPSCs for disease understanding and therapeutic development, ultimately advancing efforts toward effective treatments for ALS.

## Inclusion criteria

The publications included in this review were searched in PubMed using the following keywords: “iPSC”, “TDP43”, “TARDBP”, “MNs”. Of the over 60 papers published since 2012, we included 36 papers that studied iPSCs derived from ALS patients with *TARDBP* mutations or control iPSC lines that were edited with mutations of the *TARDBP* gene. We excluded studies working with overexpression or knock-down of TDP43 in iPSC in order to focus on more disease-relevant models. While we aimed to capture all relevant studies using the defined search strategy, it is possible that some publications were not retrieved or did not meet the inclusion criteria.

## Main text

### Differentiating iPSCs into MNs: a plethora of protocols

The first protocols to generate MNs in vitro were done using mouse embryonic stem cells [25], which laid the ground for the later-developed differentiation protocols from human iPSCs [15]. During human development, MNs are generated under the regulation by specific signaling pathways activated by specific factors at specific timepoints. This very coordinated signaling machinery has been extensively studied in amphibian, chick and mouse embryos [26]. Protocols have been developed to recapitulate MN generation in vitro, either by administering small molecules to iPSCs, or by directly activating the targeted transcription factors. In the 36 studies included in this review, 34 used small molecule-directed differentiation, while only 2 used transcription factor-directed differentiation.

#### Small molecule-directed differentiation protocols

Since 2008, more than 20 protocols have been published using small molecules to direct the differentiation of iPSCs into MNs. Although they generally follow three core developmental steps, neuralization, ventralization, and rostrocaudal patterning [27], they can be subdivided into two conceptually distinct strategies.

Early small molecule-based protocols relied primarily on exposure to retinoic acid and SHH agonists to induce spinal MN identity [25, 28, 29]. These strategies recapitulate aspects of ventral neural tube patterning but primarily produce MNs of rostral (cervical or anterior brachial) identity.

In contrast, later protocols, particularly those derived from Maury et al. [30] and Du et al. [31], incorporated an intermediate axial or neuromesodermal progenitor-like (NMP-like) stage induced by WNT signaling. By first generating WNT-responsive axial-like progenitors and then modulating the timing of retinoic acid exposure, these protocols allow controlled progression of HOX activation before neural commitment, thereby enabling specification of brachial, thoracic, or more caudal MN identities [32]. This shift represents not merely a technical refinement but a conceptual advance toward reproducing trunk developmental dynamics in vitro.

In the studies included in this review, most protocols were adapted from one of these two developmental frameworks. In practice, authors typically cited a previously established protocol as a conceptual basis and then introduced laboratory-specific modifications, including adjustments in factor concentration, timing, culture format, or maturation conditions (Table 1). As a result, while the underlying developmental logic often traces back to a limited number of reference protocols, substantial methodological variability exists across studies. In the following sections, we therefore describe the differentiation process step by step, highlighting the differences in signaling factors that may contribute to variability in MN identity, maturation state, and experimental outcomes.

**Table 1:** Studies included in the review and details on their differentiation protocol

	Author	Year	Mutations	Factors used for differentiation	Cited protocols	Day of post-mitotic MN specification	Days at analysis	Coating	Purity
1	Bilican et al. [44]	2012	M337V (M56)	SB, Dorsomorphin, N-acetylcysteine, RA, PMA, FGF, BDNF, GDNF, forskolin, DAPT	Chambers SM, et al. [45]	~ 29	~ 47–71	Lam + fibronectin	50%: HB9^+^ 20%: SMI-32^+^
2	Egawa et al. [46]	2012	G298S (M55) M337V (M64) Q343R (F62)	Dorsomorphin, SB, RA, SHH, FGF, BDNF, GDNF, NT3	T. Wada et al. [47]	~ 29	~ 60	PLL +Lam + fibronectin	60%: MAP2^+^ 10%: GFAP^+^ 30%: SMI-32^+^
3	Zhang et al. [48]	2013	A90V (F75) M337V (M56)	/	HD iPSC Consortium [49]	~ 39	~ 53	PLL + Lam	65%: Tuj1^+^
4	Alami et al. [50]	2014	G298S (M43) A315T (M70) M337V (M56)	RA, PMA, BDNF, GDNF, CNTF, NT3	Dimos et al. [51]	14	21–28	PDL + Lam	HB9-GFP sorted
5	Devlin et al. [52]	2015	M337V (M)	NAC, Activin Inhibitor, Dorsomorphin, FGF/heparin, RA, PMA, BDNF, GDNF, SAG	Amoroso et al. [28] Billican et al. [44]	~ 30–35	50–156	PLL + Lam + fibronectin + Matrigel	80%: Tuj1^+^ 50%: Hb9^+^ 20%: GFAP^+^
6	Seminary et al. [53]	2018	M337V (M56)	SB, LDN, RA, SAG, CHIR, DAPT	Maury et al. [30]	14	21–28	Matrigel	60%: Tuj1% > 95%: ChAT^+^/Tuj1^+^
7	Bossolasco et al. [54]	2018	A382T (F63)	SB, LDN, AA, RA, SAG, PMA, GDNF, CNTF	Amoroso et al. [28]	24	24	PLL + Lam	80%: SMI32^+^ 20%: HB9^+^ cells
8	Kreiter et al. [55]	2018	G294V (M46) S393L (F87)	SB, Dorsomorphin, CHIR, PMA, AA, RA, BDNF, GDNF, dbcAMP	Reinhardt et al. [56]	25	25–60	Matrigel	50%: Tuj1^+^ 100%: MAP2^+^ / Tuj1^+^ 60%: SMI-32^+^ / Tuj1
9	Fujimori et al. [57]	2018	M337V (M55) Q343R (F62)	SB, Dorsomorphin, CHIR, RA, PMA, BDNF, GDNF, AA, DAPT	/	30	20–65	PLO + Matrigel	86%: Tuj1^+^ 45%: ChAT^+^ 65%: HB9^+^ 60%: SMI-32^+^
10	Sun et al. [58]	2018	G298S (M64)	SB, CHIR, LDN, RA, PMA, compound E	Chambers et al. [45] Du et al. [31]	21	31	Lam	> 90%: Tuj1^+^ cells > 90%: HB9^+^ and CHAT^+^
11	Klim et al. [59]	2019	G298S (/) A315T (/) M337V (/) Q343R (/)	SB, LDN, RA, SAG, DAPT, SU-5402, RA, BDNF, GDNF, CNTF	/	14	24	PDL + Lam	HB9^+^ sorted
12	Fang et al. [60]	2019	G298S (M) N352S (F59, M45, M53)	AA, CHIR, PMA, SB, Dorsomorphin, SAG, BDNF, GDNF, CNTF, DAPT	Reinhardt et al. [56]	28	30	Matrigel	No quantification
13	Bursch et al. [61]	2019	G294V (M46) S393L (F87)	SB, Dorsomorphin, CHIR, PMA, AA, SAG, RA, BDNF, GDNF, dbcAMP	Naujock et al. [62]	28	32	PDL+ Lam	No quantification
14	Dafinca et al. [63]	2020	M337V (M57) I383T (M60)	AA, compound C, CHIR, RA, SAG, BDNF, GDNF, DAPT	Maury et al. [30]	28	30	Lam	80%: SMI32^+^ 75%: CHAT^+^ 90%: ChAT^+^/SMI32^+^
15	Ratti et al. [64]	2020	A382T (M26, M56, F60)	LDN, SB, RA, SHH, BDNF, GDNF, CNTF	Bossolasco et al. [54]	27	27	PDL + Lam	100%: Tuj1^+^ 80%: SMI312^+^ 20%: Hb9^+^
16	Yu et al. [65]	2020	G298S (M43) M337V (F62) A382T (M50)	CHIR, Dorsomorphin, SB, RA, PMA, VA, Compound E	Du et al. [31]	18	28	Matrigel	MNX1^+^ and ChAT^+^ (by qPCR, no %)
17	Choi et al. [66]	2020	A90V (/) M337V (/) Q343R (/)	AA, CHIR, DMH1, SB, RA, PMA, Compound E	Lopez-Gonzalez et al. [67]	18–20	32–40	Lam	No quantification
18	Fazal et al. [68]	2021	G287S (F/) N290S (M/) A382T (F/)	SB, LDN, CHIR, RA, DAPT, BDNF, CN	Maury et al. [30]	14	38	Lam	75%: ChAT^+^ 90%: Islet^+^ 70%: SMI-32^+^
19	Mitsuzawa et al. [69]	2021	M337V (F62) Q343R (M55) N345K (M63) G376D (M36)	/	Fujimori et al. [57]	19	33–40		No quantification
20	Smith et al. [70]	2021	Q331K (ed.) M337V (ed.)		Commercial (CDI iCell MNs)	/	20	PEI + Lam	No quantification
				LDN, SB, RA, SHH, BDNF, GDNF, CNTF	Amoroso et al. [28]	25–30	40–90	PLO + Lam	60%: MAP2 30%: Islet^+^
21	Dash et al. [71]	2022	G294V (M46) S393L (F85)	PMA, RA, BDNF, GDNF, dbcAMP	Reinhardt et al. [56]	20	30	/	80%–90% TuJ1^+^ 80%–90% MAP2^+^ 70%–75% SMI32^+^
22	Krach et al. [72]	2022	G298S (M43) N352S (M53)	AA, Dorsomorphin, SB, CHIR, RI, RA, SAG, BDNF, GDNF, CNTF, DAPT	Chambers et al. [45]	24	30	PDL + Lam	90%: Tuj1^+^ 50%: Islet^+^
23	Valdebenito-Maturana et al. [73]	2022	M337V (/)	/	/	28	35–42	PDL + Lam + Glial sandwich	> 70% HB9^+^
**24**	**Varderidou-Minasian et al.** [74]	**2022**	**I383T (M60)**	**LDN, CHIR, SB, AA, RA, PMA, cAMP, BDNF, GDNF, CNTF, BrainXell**	**Du et al.** [31]	**17**	**24**	**PDL +** **Lam**	**No quantification**
25	Onda-Ohto et al. [36]	2023	A382T (ed.)	CHIR, SB, Dorsomorphin, RA, PMA, BDNF, LIF, GDNF, AA, dbcAMP Lhx3, Ngn2, Isl1	**/**	20	20–60	PLO + Lam	45%: ChAT^**+**^
26	Lefebvre-Omar et al. [40]	2023	G348C (M53)	SB, LDN, CHIR, AA, RA, SAG, BDNF, GDNF, DAPT, CNTF, IGF1	Maury et al. [30]	17	17–70	PLO + Lam	80%: Islet^+^ 95%: VACHT^+^
27	Aly et al. [75]	2023	G298S (M64) N390D (M)	SB, Dorsomorphin, CHIR, PMA, AA, sodium selenite, progesterone, apotransferrin, insulin, putrescine, RA, BDNF, GDNF, IGF-1, cAMP	Catanese et al. [76]	21	42	Matrigel	No quantification
28	Held et al. [37]	2023	G298S (ed.)	GDNF, BDNF, CTNF, IGF-1, RA Lhx3, Ngn2, Isl1	Briggs et al. [77]	10	10	PEI + Lam	> 95%: HB9, CHAT, NEUN, ISL1, and TUJ1^+^
29	Catanese et al. [78]	2023	G298S (M64) N390D (M)	SB, Dorsomorphin, CHIR, PMA, AA, cAMP, sodium selenite, progesterone, apotransferrin, insulin, putrescine, RA	Catanese et al. [76]	21	28	Matrigel	No quantification
30	Harley et al. [79]	2023	G298S (M64)	CHIR, SB, LDN, RA, PMA, VA, SB, LDN,	Du et al. [31] Maury et al. [30]	26	26–54	PLO + Lam	HB9^+^ sorted
31	Lépine et al. [41]	2024	G348C (ed.) A382T (ed.)	CHIR, SB, DMH1, RA, PMN, VPA, Compound E, BDNF, CNTF, IGF-1	Du et al. [31]	21	28–63	PLO + lam	75%: HB9^+^ 60%: Islet^+^
32	Watts et al. [80]	2024	G298S (M43)	AA, SB, LDN, CHIR, RA, SAG, BDNF, DAPT, GDNF, CNTF	Maury et al. [30]	18	23	Borate buffer + PLO + Lam + Fibronectin	80%: Tuj1^+^ 30%: Tuj1^+^ Islet^+^
33	Dafinca et al. [81]	2024	M337V (M57) I383T (M60)	AA, Compound C, CHIR, RA, SAG, BDNF, GDNF, DAPT	Dafinca et al. [63]	19	30–35	Lam	No quantification
34	Schweingruber et al. [82]	2025	M337V (ed.)	SB, LDN, CHIR, AA, RA, SAG, DAPT, GDNF, BDNF	/	14	21–28	Fibronectin + PLO + Lam	> 95%: neurons 70%: MNs
35	Lépine et al. [83]	2025	G348C (ed.) A382T (ed.)	CHIR, SB, DMH1, RA, PMA, VPA, Compound E, IGF-1, BDNF, CNTF	Du et al. [31]	21	49	PLO + Lam	75%: HB9^+^ 60%: Islet^+^
36	Ma et al. [84]	2025	G298S (M64) A382T (F62)	LDN, SB, CHIR, RA, SAG, Compound E, BDNF, GDNF, CNTF	Du et al. [31]	23	28–50	PLO + Lam	> 80%: Islet^+^ > 80%: HB9^+^

For each study, the table summarizes the iPSC lines (mutations), key patterning/maturation factors used, referenced (cited) protocols, the approximate day of post-mitotic MN specification, the day(s) at which analyses were performed, substrate/coating, and reported MN purity/marker composition

Mutation notation. Mutations are indicated using protein-level nomenclature (e.g., M337V). When available, patient sex and age at biopsy are indicated in parentheses: M = male, F = female, and the number corresponds to age (years) at biopsy (e.g., M56). “ed.” indicates an isogenic gene-edited iPSC line (typically control iPSCs engineered to carry the indicated mutation)

“**/**” indicates that the information was not reported in the original study

Day of post-mitotic MN specification. This column reports the first time point at which cultures are described as post-mitotic MNs (typically based on expression of MN markers or equivalent staging terminology used by the authors). When the study did not explicitly define a post-mitotic transition, the timing is reported as approximate (** ~**) based on the authors’ staging/marker timelines

Abbreviations for coatings. Lam: laminin, PDL: poly-D-lysine, PLL: poly-L-lysine, PLO: poly-L-ornithine, PEI: polyethylenimine

Abbreviations for differentiation factors/conditions. AA: ascorbic acid, FGF: basic fibroblast growth factor, BDNF: brain-derived neurotrophic factor, cAMP / dbcAMP: cyclic AMP / dibutyryl-cAMP, CHIR: CHIR99021 (GSK3 inhibitor), CNTF: ciliary neurotrophic factor, DAPT: γ-secretase inhibitor, DMH1: BMP pathway inhibitor, GDNF: glial cell line-derived neurotrophic factor, IGF-1: insulin-like growth factor-1, LDN: LDN-193189 (BMP pathway inhibitor), LIF: leukemia inhibitory factor, NT3: neurotrophin-3, PMA: purmorphamine, PMN: purmorphamine, RA: retinoic acid, SAG: Smoothened agonist, SB: SB431542, SHH: Sonic hedgehog

Highlighted in bold: Non-peer-reviewed pre-print

A common early step in most MN differentiation protocols is the induction of a neuroectodal fate from iPSCs [15]. During embryonic development, formation of the neural plate requires inhibition of TGF-β and BMP signaling, preventing mesodermal and endodermal specification and promoting neural identity. In vitro, this is typically achieved through inhibition of TGF-β signaling with SB431542 and BMP signaling with either Dorsomorphin or its more specific analog LDN193189, a strategy commonly referred to as dual SMAD inhibition. In the studies included in this review, dual SMAD inhibition was performed with SB431542 + Dorsomorphin in 11 studies and SB431542 + LDN193189 in 9 studies (Table 1). Even though studies have shown that LDN193189 exhibits greater specificity for BMP receptors than Dorsomorphin [33], this factor is still used in recent papers to induce neural induction in iPSCs.

Following neural induction, ventralization is achieved by activation of Sonic Hedgehog (SHH) signaling, which specifies ventral neural tube identity and induces expression of MN progenitor markers such as OLIG2. In vitro, this is accomplished using SHH itself or small-molecule agonists such as Smoothened agonist (SAG) and Purmorphamine (PMA), either alone or in combination. In the reviewed papers, only 3 studies used SHH itself, 5 papers used SAG, 11 papers used PMA and 5 papers used a combination of SAG and PMA.

Rostrocaudal specification is then established through modulation of WNT and retinoic acid signaling. CHIR-99021 (CHIR), a GSK3 inhibitor that activates canonical WNT signaling, is commonly used to promote posterior identity, often in combination with retinoic acid to confer spinal cord characteristics. In the included studies, 9 protocols used retinoic acid alone, typically following more classical patterning approaches. In contrast, the majority combined retinoic acid and CHIR, reflecting strategies that incorporate a WNT-responsive axial or neuromesodermal progenitor-like stage prior to motor neuron specification, thereby allowing more controlled caudalization and modulation of HOX gene activation dynamics.

The final step in all MN differentiation protocols is maturation. In vitro, this is first obtained by treatment with neurotrophic growth factors that promote neurite growth and neuronal survival, such as BDNF (brain-derived neurotrophic factor), GDNF (glial cell derived neurotrophic factor), CNTF (ciliary neurotrophic factor) and IGF-1 (insulin-like growth factor 1). Secondly, maturation is also obtained by inhibiting NOTCH signaling, which promotes proliferation of neural progenitors, with factors such as DAPT and compound E. At this step, there is considerable variability in the composition and duration of maturation cocktails across studies.

Although there seems to be a consensus on the small molecules used to differentiate MNs, substantial heterogeneity exists in the combination, concentration, and timing of these factors. We identified more than 27 distinct referenced protocols across the 36 studies included in this review, with most laboratories introducing protocol-specific modifications. Importantly, the duration of differentiation also varies markedly. To address this variability, we distinguished in Table 1 between (1) the day of post-mitotic MN specification and (2) the day(s) at which experiments were performed. Across studies, post-mitotic specification occurs between approximately day 10 and day 35, whereas analyses were conducted anywhere from ~ 20 days to more than 70 days in culture. This broad temporal window likely reflects differences not only in maturation state but also in the degree of in vitro “aging” of the derived MNs. As neuronal maturity influences electrophysiological properties [34] and metabolic profile [35] among other parameters, differences in culture duration may substantially contribute to the discrepancies in phenotypic severity reported across studies.

Moreover, most of the studies included in this review have made their own modifications to the cited protocols, adding even more to the heterogeneity. One might have expected that, over time, protocols yielding the highest MN purity and reproducibility would become standardized across laboratories. Instead, due to the multiple efficient approaches available to induce MN fate, most laboratories tend to adopt and adapt protocols that best suit their technical environment, desired yield, experimental timeline, and local expertise.

#### Transcription factor-directed MN differentiation

During development, signaling factors lead downstream to the activation of transcription factors that are the ones directly responsible for MN differentiation and specification. Small molecule-based protocols aim to recapitulate these developmental signaling cascades, thereby mimicking embryonic patterning events. However, because transcription factors are the final effectors of lineage specification, directly expressing these factors can be sufficient to induce MN identity and may reduce the variability and heterogeneity in MN yield sometimes observed with small molecule approaches [27].

Importantly, transcription factor-directed strategies are not intended to replace developmental patterning approaches but rather represent an alternative method with distinct advantages, including shorter differentiation timelines and increased cellular homogeneity. Nevertheless, forced overexpression does not fully reproduce endogenous developmental dynamics and may introduce artificial aspects of gene regulation.

In our review, only 2 studies preferentially used transcription factor-directed MN differentiation. This limited number likely reflects the additional technical requirements of these protocols, which involve either genetic integration of transcription factor constructs at the iPSC stage or viral delivery into neural progenitors. In Onda-Ohto et al. [36], an adenovirus vector expressing NGN2, ISL1 and LHX3 was added to iPSC-derived neural progenitors and led to a purity of around 50% MNs in the obtained population. In Held et al. [37], PiggyBac plasmids with the same three tetracycline inducible transcription factors (NGN2, ISL1, and LXH3) were directly integrated at the iPSC stage, and after 13 days of differentiation, this led to a very high purity of MN population with more than 95% of the cells expressing MN specific markers.

#### Purity of iPSC-derived MNs

To conclude on the purity of the obtained MN population, it is routine in most of the published studies to quantify different markers of MNs on iPSC-derived MNs using immunocytochemistry. Tuj1, labelling β-III tubulin, and MAP2 (microtubule-associated protein 2), are commonly used as general neuronal markers to identify the neuronal fate of the obtained population. To identify more specifically motor neuron identity, three main markers are used: Islet (ISL1/ISL2—Islet LIM Homeobox 1/2) as an early-stage motor neuron marker; ChAT (Choline Acetyltransferase) as a marker of functional cholinergic MNs; and HB9 (MNX1—Motor Neuron and Pancreas Homeobox 1) as a marker of mature MNs [38]. NF antibodies are also commonly used, including SMI-32, which labels non-phosphorylated neurofilament-H, and SMI-312, which recognizes phosphorylated NF epitopes, to identify large projecting neurons, including MNs, but it is not exclusive to MNs.

Although these markers are widely used, antibody specificity, clone selection, and staining protocols vary across laboratories and can affect marker detection and quantification. HB9/MNX1, for example, is a well-established transcription factor required for MN specification and maintenance during development. However, its expression can be developmentally regulated and is context-dependent [39]. As a result, HB9 levels may vary depending on the maturation stage of the neurons and the culture conditions, potentially leading to under- or overestimation of MN purity when used as a sole marker. This stage-dependent expression complicates direct comparison of HB9-positive percentages across studies using different differentiation timelines or different time points of analysis.

Despite variability in marker selection and detection methods, a general trend can be identified on which protocols achieved the highest reported purity of MNs. The Held et al. [37] protocol appears to be among the most efficient, producing > 95% pure MNs in just 13 days using transcription factor-directed differentiation. Using small molecules-directed differentiation, Lefebvre-Omar et al. [40] and Lépine et al. [41] also achieved very high purities.

Since in ALS different MN subtypes show varying degrees of vulnerability to disease onset and progression, with the example of ocular MNs remaining relatively resistant throughout disease progression [42], it is thus crucial o define which MN subtypes are generated by current differentiation protocols. Importantly, both upper corticospinal MNs (in the brain) and lower somatic MNs degenerate in ALS. However, the protocols discussed in this review, focus exclusively on generating lower MNs. This reflects the current technical challenges associated with generating defined subclasses of upper MNs in vitro [15].

The advent of transcriptomics analysis now allows researchers to better characterize the specific MN subtypes generated from iPSCs. Lefebvre-Omar et al. [40] used transcriptomics to confirm that their protocol produced cervical cholinergic skeletal MNs, confirmed by expression of ISLET1 (ISL1) and HB9 (MNX1), while cholinergic motor neuron identity was supported by expression of *ACHE* (acetylcholinesterase) and *VACHT* (vesicular acetylcholine transporter). Importantly, they further demonstrated that these neurons predominantly correspond to alpha MNs, as distinguished from gamma MNs based on the differential expression of *RBFOX3* (RNA binding fox-1 homolog 3), *SV2B* (synaptic vesicle glycoprotein 2B), and *STK32A* (Serine/threonine-protein kinase 32A). The authors suggest that these MNs could specifically innervate limb muscles, validating their use for ALS modelling. Similarly, Ziff et al. [43] integrated transcriptomic data from 18 iPSC-derived MN studies, including two involving *TARDBP* patient-derived cells, and found that all protocols primarily generated hindbrain, cervical, and thoracic MNs, but not lumbar or sacral MNs. These findings indicate that current differentiation protocols tend to favour the generation of cervical MNs, highlighting a gap in the ability to model ALS in neurons innervating lower-body muscles.

### *TARDBP* mutations included in iPSC modelling studies

**Fig. 1:**
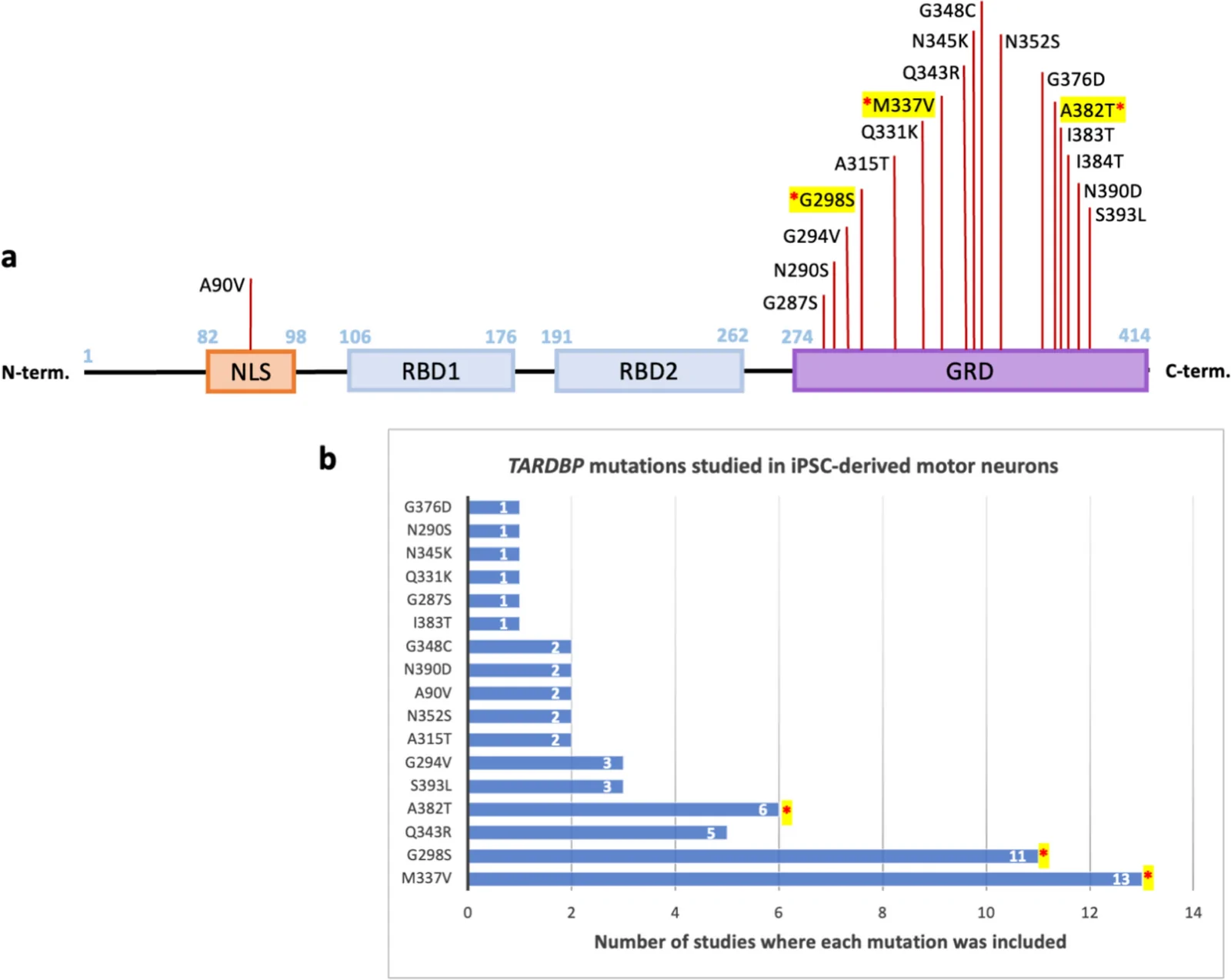
Domain organization of TARDBP  and representation of its mutations in iPSC-derived motor neuron studies. **a** Linear schematic of human TARDBP gene (414 aa) showing the nuclear localization signal (NLS), RNA recognition motifs (RRM1 and RRM2), and the C-terminal glycine-rich/low-complexity domain (GRD/LCD). ALS-associated *TARDBP* mutations discussed in this review are mapped to their respective amino acid positions. Most mutations cluster within the C-terminal glycine-rich domain. Frequently studied mutations are highlighted. **b** Number of independent studies in which each mutation was modeled in iPSC-derived MNs among the 34 publications included in this review

More than 40 different missense mutations have been identified on *TARDBP*, most of which are disease-causative [21]. These mutations are considered to cause fALS, but they also have been identified in sporadic ALS (sALS) patients with no family history. *TARDBP* mutation carriers represent approximately 2% to 5% of familial and 1.5% of sALS cases [21]. Mutations in *TARDBP* are massively clustered in the C-terminal region of the gene encoding for the glycine-rich domain of the TDP-43 protein, known to be involved in protein–protein interactions [85]. The most frequent ALS variants of TDP-43 in patients are A382T, M337V and G348C [86], 2 of which have been thoroughly studied using iPSC modelling. The M337V-iPSC line has been the most studied in the TDP-43-iPSC modelling field. This cell line was also the first one to be differentiated into MNs and characterized [44] (Fig. 1).

### Phenotypes of *TARDBP* iPSC-MNs

#### Morphology of *TARDBP* mutant iPSC-MNs

The understanding of ALS pathophysiology is challenged by the fact that the degenerating MNs are located in the brain and spinal cord of patients, making them inaccessible for studying during disease progression. Analysing post-mortem tissues has been informative for elucidating pathways of degeneration and disease; however, at this stage only the remaining MNs can be characterized. Thus, there is no information on MN morphology and characteristics during early disease development in ALS patients. The iPSC-derived MNs from ALS patients offer a valuable tool to study the earliest stage of ALS pathogenesis, before fatal degeneration.

Concerning the general morphology of MNs derived from iPSCs, Kreiter et al. [55] reported that *TARDBP* MNs exhibit higher percentages of beady and disrupted neurites compared to control MNs. Similarly, Fujimori et al. [57] reported an increased percentage of *TARDBP* MNs with neurite swelling compared to controls. On the contrary, other studies have reported unaltering of morphology of *TARDBP* MNs; however, they reached this conclusion without any quantification (Zhang et al. [48]; Onda-Ohto et al. [36]) or by quantifying other parameters such as the number of neurites per neuron [48] (Table 2).

**Table 2:** Neurite length and morphology alterations in *TARDBP* mutant motor neurons

Author	Year	Mutations	Coating	Purity	Day of post-mitotic MN specification	Days at analysis	Phenotype
Egawa et al. [46]	2012	G298S (M55) M337V (M64) Q343R (F62)	PLL + Lam + fibronectin	60%: MAP2+ 10%: GFAP+ 30%: SMI-32+	~ 29	~ 38	*Length: Decreased*
Zhang et al. [48]	2013	A90V (F75) M337V (M56)	PLL + Lam	65%: Tuj1+	~ 39	~ 53	**Morphology: similar**
							**Neurites per cell: similar**
Kreiter et al. [55]	2018	G294V (M46) S393L (F87)	Matrigel	50%: Tuj1+ 100%: MAP2+ / Tuj1+ 60%: SMI-32+ / Tuj1	~ 25	~ 30–57	*Morphology: altered (disrupted neurites)*
Fujimori et al. [57]	2018	M337V (M55) Q343R (F62)	PLO + Matrigel	86%: Tuj1+ 45%: ChAT+ 65%: HB9+ 60%: SMI-32+	30	20–65	*Length: Decreased*
							*Morphology: altered (swelling)*
Mitsuzawa et al. [69]	2021	M337V (F62) Q343R (M55) N345K (M63) G376D (M36)	/	/	~ 19	~ 33–40	*Length: Decreased*
							*Branching: Increased*
Onda-Ohto et al. [36]	2023	A382T (ed.)	PLO + Lam	45%: ChAT+	~ 20	~ 40	**Morphology: similar**
Lépine et al. [41]	2024	G348C (ed.) A382T (ed.)	PLO + Lam	75%: HB9+ 60%: Islet+	21	28–63	**Axonal area: similar** **Axonal branching: similar**

Summary of studies assessing survival or viability of iPSC-MNs carrying *TARDBP* mutations. For each study, the mutation analyzed, differentiation protocol, coating conditions, neuronal purity, timing of post-mitotic MN specification, and day of analysis are indicated.  Italic bars indicate studies reporting altered morphological properties in *TARDBP*-mutant MNs compared to controls, whereas bold bars indicate no detectable differences.

Mutation notation. Mutations are indicated using protein-level nomenclature (e.g., M337V). When available, patient sex and age at biopsy are indicated in parentheses: M = male, F = female, and the number corresponds to age (years) at biopsy (e.g., M56). “ed.” indicates an isogenic gene-edited iPSC line (typically control iPSCs engineered to carry the indicated mutation)

“/” indicates that the information was not reported in the original study

Abbreviations: LDH, lactate dehydrogenase; CC3, cleaved caspase-3; DSB, double-strand breaks

Focusing more closely on neurite length in MNs derived from *TARDBP* ALS patients, only three studies have reported alterations. Egawa et al. [46] showed decreased neurite length in HB9-GFP-expressing *TARDBP* MNs compared to controls. Fujimori et al. [57] reported similar neurite length to controls until day 40; which thereafter decreased in the *TARDBP* MNs but not in control lines. Mitsuzawa et al. [69] investigated the length of neurites at the edge of an undissociated MN embryoid body, where *TARDBP* MNs showed decreased neurite length, but increased axonal branching (Table 2). Interestingly, this motor neuron phenotype has been observed in other in vivo models of TDP-43. Overexpression of TDP-43 mutations in zebrafish larvae led to decreased axonal length but increased axonal branching of MNs [87, 88]. Similar results were observed when overexpressing TDP-43 mutations in chick embryos [89]. However, in a recent study using iPSC-derived MNs, Lépine et al. [41] reported that both the axonal area and the axonal branching were similar between control lines and two *TARDBP*-mutated lines.

In conclusion, only a few of the 36 studies detected morphological changes related to neurite morphology and length. Importantly, the decreased neurite length detected in some studies, which correlated with neurite swelling, appears to be closely related to the general cell death of *TARDBP* patients’ MNs in culture. Taken together, discrepancies across studies and quantification methods indicate that this phenotype is variable and strongly influenced by the conditions under which MNs extend neurites. To reach definitive conclusions about potential alterations, future work should evaluate these parameters using microfluidic devices in a standardized, reproducible context.

#### Cell death and degeneration of *TARDBP* mutant iPSC-derived MNs

The iPSC-derived MNs from ALS patients enable the determination of cell-autonomous, intrinsic characteristics of MNs that could drive their degeneration. The following paragraph summarizes studies of baseline cell viability in MNs derived from *TARDBP* patients to assess whether *TARDBP* mutations alone are sufficient to cause MN degeneration (Table 3).

**Table 3:** Viability of *TARDBP* mutant iPSC-derived motor neurons

Author	Year	Mutations	Coating	Purity	Day of post-mitotic MN specification	Days at analysis	Viability	Method
Bilican et al. [44]	2012	M337V (M56)	Laminin Fibronectin	50%: HB9+ 20%: SMI-32+	~ 29	~ 47–71	*Altered*	Automated fluorescence microscopy
Egawa et al. [46]	2012	G298S (M55) M337V (M64) Q343R (F62)	PLL Laminin Fibronectin	60%: MAP2+ 10%: GFAP+ 30%: SMI-32+	~ 29	~ 60	**Unaltered**	LDH release
Devlin et al. [52]	2015	M337V (M)	PLL Laminin Fibronectin Matrigel	80%: Tuj1+ 50%: Hb9+ 20%: GFAP+	~ 30–35	50–156	**Unaltered**	Cell count Pyknotic nuclei count LDH leakage
Seminary et al. [53]	2018	M337V (M56)	Matrigel	60%: Tuj1+ > 95%: ChAT+ /Tuj1+	14	21–28	**Unaltered**	Cell count
Kreiter et al. [55]	2018	G294V (M46) S393L (F87)	Matrigel	50%: Tuj1+ 100%: MAP2+ / Tuj1+ 60%: SMI-32+ / Tuj1+	25	25–60	Depends on method and mutation type	Pyknotic cell nuclei LDH leakage Hoechst nuclei counting DNA damage / DSB
Fujimori et al. [57]	2018	M337V (M55) Q343R (F62)	PLO Matrigel	86%: Tuj1+ 45%: ChAT+ 65%: HB9+ 60%: SMI-32+	30	20–65	*Altered*	LDH leakage CC3 staining
Yu et al. [65]	2020	G298S (M43) M337V (F62) A382T (M50)	Matrigel	MNX1+ and ChAT+ (by qPCR, no %)	18	28	*Altered*	LDH leakage
Lépine et al. [41]	2024	G348C (ed.) A382T (ed.)	PLO + Lam	75%: HB9+ 60%: Islet+	21	28–63	**Unaltered**	ATP-based luminescent viability assay CC3 staining

Summary of studies assessing survival or viability of iPSC-MNs carrying *TARDBP* mutations. For each study, the mutation analyzed, differentiation protocol, coating conditions, neuronal purity, timing of post-mitotic MN specification, and day of analysis are indicated. In Kreiter et al., viability outcomes varied depending on the mutation and the assay used. Italic bars indicate studies reporting altered viability in *TARDBP*-mutant MNs compared to controls, whereas bold bars indicate no detectable differences.

Mutation notation. Mutations are indicated using protein-level nomenclature (e.g., M337V). When available, patient sex and age at biopsy are indicated in parentheses: M = male, F = female, and the number corresponds to age (years) at biopsy (e.g., M56). “ed.” indicates an isogenic gene-edited iPSC line (typically control iPSCs engineered to carry the indicated mutation)

Abbreviations: LDH, lactate dehydrogenase; CC3, cleaved caspase-3; DSB, double-strand breaks

Several studies have reported decreased viability of *TARDBP* MNs compared to control lines. In the study by Bilican et al. [44], automated fluorescence microscopy was used to track the loss of fluorescence in HB9-GFP–transfected cells, revealing reduced viability of *TARDBP* MNs relative to controls. In Fujimori et al. [57] and Yu et al. [65], *TARDBP* lines exhibited increased lactate dehydrogenase (LDH) release into the extracellular medium compared with controls, indicating cytotoxicity and, more specifically, loss of cell membrane integrity. Furthermore, Fujimori et al. [57] reported increased staining for cleaved caspase-3 in *TARDBP* MNs, suggesting that loss of *TARDBP* MNs is related to apoptotic pathways*.*

Conversely, other studies found no effect on the viability of *TARDBP* MNs. In Egawa et al. [46], LDH leakage assays showed no difference between control and *TARDBP* MNs. Devlin et al. [52] monitored viability under basal conditions for 10 weeks after plating using several techniques; cell counts, LDH leakage, and pyknotic nuclei quantification all showed no difference between *TARDBP* patient MNs and controls. Seminary et al. [53] measured viability 3 and 4 weeks after differentiation by counting cells and likewise detected no difference between TDP-43 and control MNs. In Lépine et al. [41], an ATP-based viability assay showed no difference between *TARDBP* mutant and control lines over 6 weeks of monitoring. Notably, when the iPSC-derived MNs were cultured without neurotrophic factors, the viability declined markedly over time in culture; however, even under these conditions, there was no difference between patient and control MNs.

Interestingly, Kreiter et al. [55] found no difference between mutant and control MNs by LDH release or nuclei counts and detected no signs of DNA damage or double-strand breaks. In contrast, the same study reported increased total cell death based on pyknotic nuclei counts in *TARDBP* lines, but this effect was mutation-specific: it was observed in the TDP-43 G294V line but not in S393L (Table 3).

Overall, there is no consensus on the in vitro baseline viability of *TARDBP* MNs. We attempted to relate the observed discrepancies to differences in the specific *TARDBP* mutations studied, MN purity, substrate coating, maturation/aging time, and quantification methods but found no clear explanation. For example, Bilican et al. [44] and Seminary et al. [53] reported divergent viability results despite using the same *TARDBP* M337V iPSC line; however, they employed different viability quantification techniques and distinct differentiation protocols to generate MNs. A likely explanation for the divergent findings is the methodological and contextual heterogeneity. These studies used non-equivalent readouts of “viability” (LDH leakage, ATP-based assays for metabolic activity, cleaved-caspase-3 for apoptosis, and pyknotic nuclei counts), which capture different stages of cell death. Moreover, the phenotypes appear in a time-dependent manner (e.g., the changes emerging after 40 days of maturation in Fujimori et al. [57]) so differences in the sampling windows can shift outcomes. Together, these factors underlie the lack of consensus on the baseline *TARDBP* MN viability. Standardized, multi-parametric assays at defined time points will be needed to resolve these discrepancies.

ALS is often a late-onset disease with the mean age of onset varying from 50 to 65 years and the median being at 64 years [90]. Why MNs start degenerating only at later age has still yet to be understood. Accumulating evidence suggests that MNs are particularly vulnerable to various cellular stressors, including oxidative stress, ER stress, excitotoxicity, and proteasome dysfunction [91–93]. Several studies have examined the viability of the *TARDBP* ALS patient-derived MNs under stress conditions in vitro (Table 4). Unlike the findings at basal state, where there is some debate, this aspect of the literature is more consistent, with *TARDBP* MNs consistently exhibiting increased vulnerability to stress compared to control lines.

**Table 4:** Viability of *TARDBP* mutated MNs after induction of stress

Author	Year	Mutations	Method to assess cell death	Pathway	Viability	Drug	Concentration	Time
Billican et al. [44]	2012	M337V (M56)	LDH leakage	MAPK inhibition	**Similar**	U0126	10 μM	48 h
				PI3K inhibitor	*Premature death of TARDBP MNs*	LY294002	40 μM	48 h
				ER stress	**Similar**	Tunicamycin	1 mg/mL	48 h
Egawa et al. [46]	2012	G298S (M55) M337V (M64) Q343R (F62)	EthD-1 staining	Oxidative stress	*Premature death of TARDBP MNs*	Sodium arsenite	500 μM	1 h
Zhang et al. [48]	2013	A90V (F75) M337V (M56)	MTT assay	Oxidative stress	**Similar**	Sorbitol	0.4 M	30 min
				Oxidative stress	**Similar**	Sodium arsenite	1–5 μM	24 h
				Oxidative stress	**Similar**	tBOOH	1–10-100 μM	24 h
				Apoptosis induction	*Premature death of TARDBP MNs*	Staurosporine	10–50-100 nM	24 h
Sun et al. [58]	2018	G298S (M64)	LDH leakage CC3 staining	Proteasome inhibition	*Premature death of TARDBP MNs*	MG132	0.6 μM	24 h
Onda-Ohto et al. [36]	2023	A382T (ed.)	CC3 staining	Oxidative stress	*Premature death of TARDBP MNs*	H_2_O_2_	25 μM	1.5 h
Watts et al. [80]	2024	G298S (M43)	Isl1/2 staining	ER stress	*Decreased viability of TARDBP MNs*	Thapsigargin	1 μM	48 h
				ER stress	*Decreased viability of TARDBP MNs*	Tunicamycin	1 μM	48 h
Dafinca et al. [81]	2024	M337V (M57) I383T (M60)	Cell proliferation test	ER stress	*Decreased viability of TARDBP MNs*	Thapsigargin	1 μM	24 h
Lépine et al. [41]	2024	G348C (ed.) A382T (ed.)	ATP-based luminescent viability assay	Excitotoxicity	*Decreased viability of TARDBP MNs*	Glutamate	0.1 mM	24 h
				Oxidative stress	*Decreased viability of TARDBP MNs* *only 1 of the 2 TARDBP lines	Ethacrynic acid	50 μM	17 h

Summary of studies evaluating the susceptibility of iPSC-MNs carrying *TARDBP* mutations to different cellular stressors. For each study, the mutation analyzed, assay used to assess cell death, stress pathway targeted, compound applied, concentration, and treatment duration are indicated

Mutation notation. Mutations are indicated using protein-level nomenclature (e.g., M337V). When available, patient sex and age at biopsy are indicated in parentheses: M = male, F = female, and the number corresponds to age (years) at biopsy (e.g., M56). “ed.” indicates an isogenic gene-edited iPSC line (typically control iPSCs engineered to carry the indicated mutation)

Abbreviations: LDH, lactate dehydrogenase; CC3, cleaved caspase-3; MTT, methylthiazolyldiphenyl-tetrazolium bromide; ER, endoplasmic reticulum; MAPK, mitogen-activated protein kinase; PI3K, phosphoinositide 3-kinase

In Bilican et al. [44], LDH assays showed that MAPK inhibition (U0126) and ER stress (tunicamycin) caused similar death across lines, whereas PI3K inhibition (LY294002) reduced *TARDBP* MN viability more than controls. Zhang et al. [48] observed no group differences with oxidative stress (sodium arsenite, tBOOH, sorbitol), but *TARDBP* MNs exhibited greater death after staurosporine-induced apoptosis by MTT. Egawa et al. [46] reported lower viability of *TARDBP* MNs after acute sodium arsenite exposure, quantified by EthD-1. Consistently, Onda-Ohto et al. [36] found significantly increased cleaved-caspase-3 after only 1.5 h of hydrogen peroxide. Additional studies reinforce this heightened susceptibility: Sun et al. [58] showed premature death after proteasome inhibition (MG132) by LDH and cleaved-caspase-3; Dafinca et al. [81] and Watts et al. [80] reported reduced viability with thapsigargin-induced ER stress, and Watts et al. [80] also confirmed the effect with tunicamycin; Lépine et al. [41] demonstrated increased mortality with glutamate-induced excitotoxicity, while ethacrynic-acid–induced oxidative stress increased mortality in only one of two *TARDBP* lines.

Taken together, it is striking that all studies identified an increased stress sensitivity of *TARDBP* MNs, even though the specific triggers differ. These differences most likely arise from the concentration of each stressor and the exposure paradigm (e.g., acute versus prolonged treatment and the timing of measurement), which can engage distinct stress response programs. In addition, the assays used to quantify injury (LDH release, ATP, EthD-1 labelling, cleaved-caspase-3) capture different stages of damage and vary in sensitivity, further contributing to divergent readouts. Standardizing stressor concentrations and exposure windows, and employing harmonized, multiparametric readouts, should help reconcile these discrepancies. Nonetheless, the heightened stress sensitivity of *TARDBP*-mutant MNs is consistently observed across studies, establishing it as a robust in vitro phenotypic readout.

#### Functionality at the electrophysiological level

Studying the electrophysiology of iPSC-derived MNs from *TARDBP* MNs in vitro is essential for understanding ALS mechanisms, as early functional changes have been observed both in human patients and in animal models of ALS. In vivo studies show that MN hyperexcitability, often linked to altered sodium currents, occurs even before symptoms appear; however, these studies are mostly conducted on the SOD1 mouse model [94]. The iPSC-derived MNs from *TARDBP* patients provide a valuable tool to investigate these early disruptions in a human context, and are related to TDP-43-ALS specifically (Table 5).

**Table 5:** Electrophysiological properties of *TARDBP* mutant motor neurons

Author	Year	Mutations	Method	Coating	Purity	Day of post-mitotic MN specification	Days at analysis	Phenotype
Smith et al. [70]	2021	Q331K (ed.) M337V (ed.)	MEA	PLO + Lam	60%: MAP2+ 30%: Islet+	25–30	40–90	*Fewer bursts* *Increased burst duration* *Increased mean firing rate*
Lépine et al. [41]	2024	G348C (ed.) A382T (ed.)	MEA	PLO + Lam	75%: HB9+ 60%: Islet+	21	28–63	**Unchanged burst properties**
								*Fewer active electrodes* *Decline in mean firing rate*
Bursch et al. [61]	2019	G294V (F67) S393L (M46)	Calcium imaging	PLO + Lam	No quantification	28	32	**No differences in spontaneous Ca**^**2+**^** transients**
								*Increased basal intracellular Ca*^*2*+^ *levels* *Higher Ca*^*2*+^ *transient amplitudes upon AMPA receptor stimulation*
Dafinca et al. [63]	2020	M337V (M57) I383T (M60)	Calcium imaging	Lam	80%: SMI32+ 75%: CHAT+ 90%: ChAT+ /SMI32+	28	30	*Lower Ca*^*2*+^ *amplitudes upon depolarization* *Longer recovery times after KCl treatment* *Increased peak amplitudes after glutamate stimulation*
Zhang et al. [48]	2013	A90V (F75) M337V (M56)	Patch-clamp	PLL + Lam	65%: Tuj1+	~ 39	~ 53	**Unchanged amplitude of post-synaptic currents**
								*Decreased spontaneous synaptic currents with lower frequency*
Devlin et al. [52]	2015	M337V (M)	Patch-clamp	PLL + Lam + Fibronectin + Matrigel	80%: Tuj1+ 50%: Hb9+ 20%: GFAP+	~ 30–35	50–156	**No changes in whole-cell capacitance or input resistance**
								*More depolarized resting membrane potentials* ***Early on****: increased hyperexcitability* ***With maturation****: progressive loss of action potential output and synaptic activity*
Kreiter et al. [55]	2018	G294V (M46) S393L (F87)	Patch-clamp	Matrigel	50%: Tuj1+ 100%: MAP2+ / Tuj1+ 60%: SMI-32+ / Tuj1+	25	25–60	**Normal sodium and potassium currents** **Normal single and repetitive action potentials** **Normal spontaneous postsynaptic currents**
Fazal et al. [68]	2021	G287S (F/) N290S (M/) A382T (F/)	Patch-clamp	Lam	75%: ChAT+ 90%: Islet+ 70%: SMI-32+	14	38	**Normal sodium and potassium currents** **Normal ability to fire spontaneous and evoked action potentials**
Smith et al. [70]	2021	Q331K (ed.) M337V (ed.)	Patch-clamp	PLO + Lam	60%: MAP2+ 30%: Islet+	25–30	40–90	*More depolarized membrane potentials* *Progressive decline in repetitive action potential firing with time in culture*
Harley et al. [79]	2023	G298S (M64)	Patch-clamp	Matrigel + Astrocytes	HB9+ sorted	26	26–54	***Early on:**** Lower action potential thresholds and increased spontaneous firing (hyperexcitability)* ***Over time:**** transition to these reduced firing frequency and impaired action potential generation (hypoexcitability)*

Summary of studies assessing electrophysiological phenotypes in iPSC-MNs carrying *TARDBP* mutations using multi-electrode array (MEA), calcium imaging, or patch-clamp recordings. For each study, the mutation analyzed, experimental method, coating conditions, neuronal purity, timing of post-mitotic MN specification, and day of analysis are indicated. Italic bars indicate studies reporting altered electrophysiological properties in *TARDBP*-mutant MNs compared with controls, whereas bold bars indicate no detectable differences

Mutation notation. Mutations are indicated using protein-level nomenclature (e.g., M337V). When available, patient sex and age at biopsy are indicated in parentheses: M = male, F = female, and the number corresponds to age (years) at biopsy (e.g., M56). “ed.” indicates an isogenic gene-edited iPSC line (typically control iPSCs engineered to carry the indicated mutation)

“/” indicates that the information was not reported in the original study

Using multielectrode array recordings, Lépine et al. [41] observed a progressive loss of spontaneous neuronal activity over time in *TARDBP* MNs, reflected by a decline in the mean firing rate and fewer active electrodes, while burst properties remained unchanged in the remaining active electrodes. Similarly, Smith et al. [70] reported fewer bursts but increased burst duration and an overall increase in the mean firing rate compared with controls, indicating altered network activity.

Calcium imaging studies further revealed disruptions in Ca^2^⁺ homeostasis. Bursch et al. [61] found increased basal intracellular Ca^2^⁺ levels in *TARDBP* MNs, with no differences in spontaneous Ca^2^⁺ transients; however, AMPA receptor stimulation elicited significantly higher Ca^2^⁺ transient amplitudes despite unchanged AMPA receptor subunit expression. In contrast, Dafinca et al. [63] reported lower Ca^2^⁺ amplitudes upon depolarization, longer recovery times after KCl treatment, and increased peak amplitudes with delayed recovery after glutamate stimulation. Blocking NMDA receptors with memantine led to elevated Ca^2^⁺ levels in *TARDBP* MNs, supporting the notion of glutamate-driven hyperexcitability.

Patch-clamp studies provided additional insight into the *TARDBP* MN dysfunction. Zhang et al. [48] observed decreased spontaneous synaptic activity, with a lower frequency but unchanged amplitude of postsynaptic currents. Devlin et al. [52] detected no changes in whole-cell capacitance or input resistance, yet *TARDBP* MNs displayed more depolarized resting membrane potentials, early hyperexcitability, and a progressive loss of action potential output and synaptic activity with maturation, linked to an age-dependent reduction in sodium and potassium currents. Consistent with this, Smith et al. [70] reported more depolarized resting membrane potentials (but only in one *TARDBP* line) and a progressive decline in repetitive action potential firing over time in culture. In contrast, Kreiter et al. [55] and Fazal et al. [68] found no significant electrophysiological alterations, with maintenance of sodium and potassium currents, action potential generation, and synaptic activity.

Harley et al. [79] provided evidence for dynamic, time-dependent changes in excitability. Early in development, *TARDBP* MNs exhibited increased axon initial segment (AIS) length, impaired AIS plasticity, and intrinsic hyperexcitability, resulting in lower action potential thresholds and increased spontaneous firing; this hyperactivity was associated with spontaneous myofiber contractions resembling ALS fasciculations. Over time, these neurons transitioned to hypoexcitability, characterized by AIS shortening, reduced firing frequency, and impaired action potential generation, linked to dysregulation of ankyrin-G and voltage-gated sodium channels.

Overall, the electrophysiological studies of *TARDBP* MNs revealed a complex, time-dependent disruption in neuronal function. Early alterations manifest as increased excitability, particularly in patch-clamp and calcium imaging studies, with hyperactive responses to stimulation and increased intracellular Ca^2^⁺ signaling. However, this initial hyperactivity is not universally observed, as some studies reported no significant deviations in sodium and potassium currents or action potential properties. Over time, the excitability of *TARDBP* MNs declines, transitioning toward hypoexcitability and synaptic dysfunction. Multielectrode array studies highlight a progressive loss of spontaneous network activity, with a reduction in burst frequency and overall neuronal output. Whole-cell patch-clamp recordings further corroborate this shift, showing a gradual loss of action potential generation and decreased ion channel conductance. While some studies report preserved neuronal function, most findings support a model in which *TARDBP* mutations lead to early hyperactivity, which later deteriorates into hypoexcitability, contributing to ALS pathophysiology. Importantly, the maturation and prolonged maintenance of MNs in vitro may also influence electrophysiological properties. As neurons age in culture, intrinsic membrane properties, synaptic connectivity, and network activity can change independently of disease mutations [34], which may contribute to the variability observed across studies.

### Does this model recapitulate ALS pathology in vitro?

#### TDP-43 aggregation and mislocalization

The iPSC-derived MNs from ALS patients provide a disease-relevant model to assess whether *TARDBP* mutations alter TDP-43 gene or protein expression in patient cells. At the mRNA level (Table 6), most studies report similar abundance of TARDBP/TDP-43 transcript between *TARDBP*-mutant and control MNs [36, 41, 44, 48, 59]. Only Egawa et al. [46] observed increased *TARDBP* mRNA by qPCR in patient MN lines; notably, there was substantial clonal variation within the same patient line, with some clones resembling controls and others showing elevated expression.

**Table 6:** *TARDBP* mRNA expression in *TARDBP* mutant motor neurons

Author	Mutations	Year	*TARDBP* expression (mRNA)
Bilican et al. [44]	M337V (M56)	2012	**Similar**
Egawa et al. [46]	G298S (M55) M337V (M64) Q343R (F62)	2012	*Increased*
Zhang et al. [48]	A90V (F75) M337V (M56)	2013	**Similar**
Klim et al. [59]	G298S (/) A315T (/) M337V (/) Q343R (/)	2019	**Similar**
Onda-Ohto et al. [36]	A382T (ed.)	2023	**Similar**
Lépine et al. [41]	G348C (ed.) A382T (ed.)	2024	**Similar**

Summary of studies evaluating *TARDBP* transcript levels in iPSC-MNs carrying *TARDBP* mutations. Reported outcomes indicate whether *TARDBP* mRNA expression is increased or unchanged compared with control motor neurons. Italic bars indicate studies reporting altered *TARDBP* RNA expression in *TARDBP*-mutant MNs compared to controls, whereas bold bars indicate no detectable differences

Mutation notation. Mutations are indicated using protein-level nomenclature (e.g., M337V). When available, patient sex and age at biopsy are indicated in parentheses: M = male, F = female, and the number corresponds to age (years) at biopsy (e.g., M56). “ed.” indicates an isogenic gene-edited iPSC line (typically control iPSCs engineered to carry the indicated mutation)

“/” indicates that the information was not reported in the original study

At the protein level (Table 7), studies have examined total TDP-43 as well as soluble and insoluble fractions. Seminary et al. [53] and Sun et al. [58] found no difference in the total TDP-43 protein between *TARDBP* mutant and control MNs, whereas Fazal et al. [68] reported increased total TDP-43 in *TARDBP* MNs. In the same study, soluble full-length TDP-43 levels were unchanged, but the insoluble fraction was significantly increased in patient MNs. Importantly, the insoluble fraction contained elevated levels of C-terminal TDP-43 fragments (CTF-35 kDa and CTF-25 kDa), which are considered pathological cleavage products associated with TDP-43 aggregation. Fazal et al. [68] also detected increased phosphorylated TDP-43 (pTDP-43) by Western blot, further supporting the presence of disease-associated TDP-43 species in mutant MNs.

**Table 7:** TDP-43 protein expression in *TARDBP* mutant motor neurons

Author	Mutations	Year	Total protein	Soluble	Insoluble	Method
Bilican et al. [44]	M337V (M56)	2012	/	*Increased*	*Increased*	RIPA-soluble fraction and detergent-insoluble pellet extracted in urea/thiourea/CHAPS
Seminary et al. [53]	M337V (M56)	2018	Similar		*Increased*	Dot-blot assay detecting insoluble TDP-43
Sun et al. [58]	G298S (M64)	2018	Similar	/	/	/
Klim et al. [59]	G298S (/) A315T (/) M337V (/) Q343R (/)	2019	/	**Similar**	**Similar**	RIPA-soluble fraction and urea-resuspended pellet; insoluble fraction loaded relative to matched soluble protein
Fazal et al. [68]	G287S (F/) N290S (M/) A382T (F/)	2021	Increased	**Similar**	*Increased*	Centrifugation-based soluble/insoluble separation
Lépine et al. [41]	G348C (ed.) A382T (ed.)	2024	Similar	**Similar**	**Similar**	RIPA-soluble fraction, and detergent-insoluble pellet extracted in urea/thiourea/CHAPS

Summary of studies evaluating TDP-43 protein levels in iPSC-derived motor neurons (MNs) carrying *TARDBP* mutations. The table reports changes in total TDP-43 protein, as well as soluble and insoluble fractions, compared with control MNs. The biochemical methods used to assess TDP-43 solubility are indicated for each study

Mutation notation. Mutations are indicated using protein-level nomenclature (e.g., M337V). When available, patient sex and age at biopsy are indicated in parentheses: M = male, F = female, and the number corresponds to age (years) at biopsy (e.g., M56). “ed.” indicates an isogenic gene-edited iPSC line (typically control iPSCs engineered to carry the indicated mutation)

“/” indicates that the information was not reported in the original study

Abbreviations: pTDP-43, phosphorylated TDP-43; RIPA, radioimmunoprecipitation assay buffer; CTF, C-terminal fragment

Similarly, Bilican et al. [44] reported increased levels of both soluble and insoluble TDP-43 in *TARDBP* patient lines, whereas Klim et al. [59] found no differences in soluble or insoluble TDP-43 levels across four TARDBP mutant lines compared with controls. Importantly, the biochemical approaches used to evaluate TDP-43 solubility differed across studies. While some protocols relied on detergent extraction followed by urea-based solubilization of the insoluble fraction and Western blot analysis (e.g., Bilican et al. [44]; Lépine et al. [41]), others applied modified workflows in which the insoluble pellet was resuspended and normalized relative to the soluble fraction (Klim et al. [59]). In addition, some studies assessed detergent-resistant TDP-43 aggregates using filter-trap assays rather than classical soluble/insoluble Western blot fractionation (Seminary et al. [53]). These methodological differences may influence the sensitivity for detecting soluble versus aggregated TDP-43 species and should be considered when comparing results across studies.

In conclusion, *TARDBP* mutations do not appear to alter gene expression at the mRNA level in patient-derived MNs, indicating no impairment of TDP-43 autoregulation. At the protein level, results are more variable across studies; however, several reports indicate an increase in insoluble TDP-43 species. Notably, only one study (Fazal et al. [68]) directly quantified pTDP-43 by Western blot, highlighting that this key pathological hallmark remains largely unexplored in iPSC-derived *TARDBP* MN models.

In ALS, cytoplasmic TDP-43 inclusions are a pathological hallmark in 98% of patients [24]. A major focus has been to understand how a predominantly nuclear protein accumulates in cytoplasmic inclusions. MNs derived from ALS patients with *TARDBP* mutations offer a relevant system to test whether *TARDBP* mutations alone can drive TDP-43 mislocalization in vitro (Table 8). In this context, 16 studies have examined subcellular distribution of TDP-43 in MNs derived from *TARDBP* patients using immunofluorescence.

**Table 8:** TDP-43 mislocalization in *TARDBP* mutant motor neurons

Author	Year	Mutations	Coating	Purity	Day of post-mitotic MN specification	Days at analysis	Technique	Mislocali-zation	Quantification
Egawa et al. [46]	2012	G298S (M55) M337V (M64) Q343R (F62)	PLL + Lam + fibronectin	60%: MAP2+ 10%: GFAP+ 30%: SMI-32+	~ 29	~ 60	IF	*Yes*	Number of aggregates
Zhang et al. [48]	2013	A90V (F75) M337V (M56)	PLL + Lam	65%: Tuj1+	~ 39	~ 53	IF	**No**	% of cells
Seminary et al. [53]	2018	M337V (M56)	Matrigel	60%: Tuj1% > 95%: ChAT+ /Tuj1+	14	21–28	IF	**No**	% of cells
Fujimori et al. [57]	2018	M337V (M55) Q343R (F62)	PLO + Matrigel	86%: Tuj1+ 45%: ChAT+ 65%: HB9+ 60%: SMI-32+	30	20–65	IF	*Yes*	Number of pTDP-43 aggregates
Bossolasco et al. [54]	2018	A382T (F63)	PLL + Lam	80%: SMI32+ 20%: HB9+ cells	24	24	IF	**No**	% of cells
Kreiter et al. [55]	2018	G294V (M46) S393L (F87)	Matrigel	50%: Tuj1+ 100%: MAP2+ 60%: SMI-32+	25	25–60	IF	**No**	No quantification
Sun et al. [58]	2018	G298S (M64)	Lam	> 90%: Tuj1+ cells > 90%: HB9+ and CHAT+	21	31	IF EM	*Yes*	No quantification
Klim et al. [59]	2019	G298S (/) A315T (/) M337V (/) Q343R (/)	PDL + Lam	HB9+ sorted	14	24	IF	**No**	TDP-43/DAPI ratio
Ratti et al. [64]	2020	A382T (M26, M56, F60)	PDL + Lam	100%: Tuj1+ 80%: SMI312+ 20%: HB9+	27	27	IF	**No**	No quantification
Dafinca et al. [63]	2020	M337V (M57) I383T (M60)	Lam	80%: SMI32+ 75%: CHAT+ 90%: ChAT+ /SMI32+	28	30	IF	**No**	% of TDP-43 in cytoplasm
Fazal et al. [68]	2021	G287S (F/) N290S (M/) A382T (F/)	Lam	75%: ChAT+ 90%: Islet+ 70%: SMI-32+	14	38	IF WB	*Yes*	N/C ratio
Smith et al. [70]	2021	Q331K (ed.) M337V (ed.)	PLO + Lam	60%: MAP2 30%: Islet+	25–30	40–90	IF	*Yes*	Intensity
Lefebvre-Omar et al. [40]	2023	G348C (M53)	PLO + Lam	80%: Islet+ 95%: VACHT+	17	17–70	IF	*Yes*	No quantification → Nuclear TDP-43 inclusions → Cytosolic pTDP-43 inclusions
Onda-Ohto et al. [36]	2023	A382T (ed.)	PLO + Lam	45%: ChAT+	20	20–60	IF	**No**	No quantification
Held et al. [37]	2023	G298S (ed.)	PEI + Lam	> 95%: HB9, CHAT, NEUN, ISL1, and TUJ1+	10	10	IF	**No**	N/C ratio
Lépine et al. [41]	2024	G348C (ed.) A382T (ed.)	PLO + lam	75%: HB9+ 60%: Islet+	21	28–63	IF WB (fractionation)	**No**	N/C ratio
Schweingruber et al. [82]	2025	M337V (ed.)	Fibronectin + PLO + Lam	> 95%: neurons 70%: MNs	14	21–28	IF	**No**	No quantification

Summary of studies assessing TDP-43 subcellular localization in iPSC-MNs carrying *TARDBP* mutations. For each study, the mutation analyzed, coating conditions, neuronal purity, timing of post-mitotic MN specification, day of analysis, detection technique, and quantification method are indicated. Italic indicates studies reporting cytoplasmic mislocalization and/or aggregation of TDP-43, whereas Bold indicates predominantly nuclear localization with no detectable differences compared with controls

An asterisk (*) indicates that phosphorylated TDP-43 (pTDP-43) was specifically assessed. When no asterisk is indicated, the analysis refers to TDP-43

Mutation notation. Mutations are indicated using protein-level nomenclature (e.g., M337V). When available, patient sex and age at biopsy are indicated in parentheses: M = male, F = female, and the number corresponds to age (years) at biopsy (e.g., M56). “ed.” indicates an isogenic gene-edited iPSC line (typically control iPSCs engineered to carry the indicated mutation)

“/” indicates that the information was not reported in the original study

Abbreviations: IF, immunofluorescence; WB, Western blot; N/C ratio, nuclear-to-cytoplasmic ratio

More than half of the studies conclude that there is no cytoplasmic mislocalization of TDP-43 in MNs derived from patients with *TARDBP* mutations. In Zhang et al. [48] and Bossolasco et al. [54], the percentage of neurons with cytoplasmic TDP-43 was 2% to 3% in both control and *TARDBP* lines. In Seminary et al. [53] around 40% of MNs in culture had cytoplasmic TDP-43-positive puncta, yet there was no significant difference in either the proportion of neurons with cytoplasmic TDP-43^+^ puncta or the number of puncta per neuron in any of the ALS iPSC lines compared to control. Klim et al. [59] quantified the TDP-43 nuclear to cytoplasmic ratio to be around 80% in both control and *TARDBP* mutant MNs, indicating predominantly nuclear localization in all lines. Lépine et al. [41] likewise found no differences in the nuclear/cytoplasmic TDP-43 by immunocytochemistry and by fractionation. Kreiter et al. [55], Ratti et al. [64], and Onda-Ohto et al. [36] reached the same conclusion of unaltered localization and absence of aggregation, although without quantification.

Conversely, other studies reported clear cytoplasmic mislocalization and/or aggregation. Fazal et al. [68] observed a decreased nuclear/cytoplasmic TDP-43 ratio in patient lines, corroborated by fractionation and immunoblotting showing higher cytosolic (but unchanged nuclear) TDP-43 in *TARDBP* MNs. Similarly, Smith et al. [70] reported increased cytoplasmic TDP-43 intensity by immunofluorescence. Egawa et al. [46] detected a slight increase in the number of cytoplasmic TDP-43 aggregates per cell in *TARDBP* MNs compared with controls. Lefebvre-Omar et al. [40] noted very rare nuclear TDP-43 dots in *TARDBP* MNs but not in controls, without quantification. Notably, Sun et al. [58] used immunoelectron microscopy and observed TDP-43 translocation to the cytoplasm and neurites in almost all *TARDBP* mutant MNs, but not in controls; MNs from sALS patients also showed extranuclear TDP-43, with fewer but larger clusters than *TARDBP* mutant lines. Importantly, TDP-43 gold particles were occasionally detected within mitochondria in *TARDBP* and sporadic MNs, but not in controls.

Given the prior evidence that TDP-43 is abnormally phosphorylated in ALS MNs [24, 95, 96], several studies have assessed pTDP-43. Fujimori et al. [57] reported a five-fold increase in the number of pTDP-43 aggregates in *TARDBP* MNs versus controls. Fazal et al. [68] described abundant somatic pTDP-43 staining (without quantitative image analysis) and detected increased pTDP-43 protein by western blot. Lefebvre-Omar et al. [40] observed very rare cytoplasmic pTDP-43 inclusions in *TARDBP* MNs but not in controls, again without quantification. In contrast, Kreiter et al. [55] did not detect pTDP-43 by immunofluorescence in either *TARDBP* or control MNs.

Overall, there is no clear consensus on TDP-43 mislocalization in iPSC-derived MNs harbouring T*ARDBP* mutations. Notably, studies that quantified the percentage of cells with mislocalized TDP-43 generally did not detect differences between patient and control MNs. Studies measuring nuclear/cytoplasmic ratios also found no differences or only small shifts. We also considered whether the cytoplasmic mislocalization of TDP-43 is age-dependent. However, we found no evidence that studies reporting mislocalization examined older neurons; across studies, there was no association between neuronal age at analysis and the presence of mislocalization. These findings suggest that, if cytoplasmic mislocalization occurs in *TARDBP*-mutant MNs, it is partial rather than total, which could explain the observed discrepancies. Importantly, TDP-43 mislocalization is not a reliable rescuable screening endpoint, given the substantial inter-study variability and the generally small effect sizes when it is observed. Another takeaway is the need for greater standardization and reproducibility in immunofluorescence studies: consistently reporting antibody references, image acquisition parameters, criteria for region-of-interest selection, and image processing workflows would enhance transparency and facilitate cross-study comparisons.

#### NF accumulation

NFs are neuron-specific cytoskeletal fibers and represent the most abundant cytoskeletal component in neurons. NF or its fragments can be released from neurons in response to axonal damage or neurodegeneration, and can subsequently be detected in the cerebrospinal fluid or blood through mechanisms that remain incompletely understood [97]. HFs can be used as biomarkers for neurodegeneration directly quantifiable in body fluids of patients. Importantly, higher levels of neurofilament light chain (NfL) and phosphorylated neurofilament heavy chain (pNfH) are associated with a higher risk of death and faster disease progression in ALS patients [98]. Moreover, NF accumulation in the perikaryon and proximal axons of the spinal cord MNs is considered a pathological hallmark of ALS [99]. Evidence also suggests that NF accumulation in ALS can also be a cause of the disease, as NF accumulation could initiate neurodegeneration by disrupting axonal transport [100, 101]. In this context, studies have investigated NF accumulation in iPSC-derived MNs from *TARDBP* patients.

In Sun et al. [58], NF aggregates accumulated significantly more in the cytoplasm and neurites of MNs from one *TARDBP* patient and two sporadic patients compared with control lines. After MN induction (day 21 of differentiation), NF aggregation was assessed at 4, 7, and 10 days in culture, showing a progressive increase in the proportion of neurons displaying NF inclusions. Electron microscopy confirmed disorganized NFs in the cytoplasm and in swollen neuritic segments and revealed mitochondria accumulation near or within NF structures. The authors hypothesized that the NF inclusions alter mitochondrial distribution, contributing to the vulnerability of patient MNs to degeneration.

In Lefebvre-Omar et al. [40], NfL accumulated significantly more in the soma of aged *TARDBP*-patient MNs than in the control line and the *TARDBP* isogenic control line. Notably, NfL accumulation occurred at earlier stages in *SOD1* and *C9orf72* patient MNs. By contrast, there was no accumulation of phosphorylated medium/heavy NF (pNF-M/H) in proximal axons of *TARDBP* MNs, whereas such accumulation was observed in *SOD1* and *C9orf72* MNs. In this study, NF accumulation was much more severe in *SOD1* and *C9orf72* patient lines, leading to axon initial segment alterations and excitability abnormalities.

Evidence from only two studies suggests NF dysregulation in *TARDBP*-mutant MNs, but the phenotype appeared modest and often age-dependent. Taken together, NF alterations are present but relatively subtle in *TARDBP* backgrounds, with subunit and compartment-specific parameters. Given the small evidence and heterogeneity, standardized, longitudinal assays are needed before adopting NF accumulation as a phenotypic readout.

#### Stress granules (SGs)

Several molecular cascades have been shown to be altered in ALS, including protein aggregation, oxidative stress and RNA dysregulation, suggesting a failure of affected MNs to cope with accumulating cellular stressors [102]. Importantly, upon various conditions of cellular stress, such as oxidative insult, heat shock and starvation, a number of key transcripts and factors, including TDP-43 and FUS, are recruited to cytoplasmic structures known as SGs [103]. SGs act as membrane-less compartments that temporarily sequester translationally stalled mRNAs, RNA-binding proteins, and the small ribosomal subunit during cellular stress, when protein translation is transiently arrested [104]. Once the stress subsides, these granules disassemble, allowing translation to resume [104]. Notably, several SG components, such as eIF3 and TIA-1, have been found to localize in aggregates in post-mortem ALS tissue [105] and *TIA-1* has been identified as a causative gene associated with fALS [106].

Several studies have examined spontaneous SG formation in iPSC-derived MNs harbouring *TARDBP* mutations. The presence of SGs at baseline could indicate upregulated stress pathways. Seminary et al. [53] stained MNs for the SG marker G3BP and observed an average of 4 G3BP-positive puncta per neuron, with no differences between control and TARDBP lines; the G3BP protein levels were likewise unchanged. In contrast, Fujimori et al. [57] reported increased numbers of G3BP-positive SGs at baseline in *TARDBP-*mutant neurons, with a disproportionate increase in SGs co-localizing with TDP-43.

To better interrogate SG dynamics, other studies induced SGs with defined stressors, enabling more controlled modelling of SG biology and the role of TDP-43 in ALS pathophysiology. Fang et al. [60] found that puromycin induced the most robust SG formation in post-mitotic iPSC-derived MNs. Using puromycin induction followed by washout, they observed similar SG formation and dissolution kinetics across genotypes; however, during recovery, significantly more TDP-43 (often co-localized with G3BP1) persisted as cytoplasmic puncta in *TARDBP* mutants than in controls. Ratti et al. [64] used sodium arsenite: acute exposure produced TIAR-positive SGs in all neurons without genotype differences, and TDP-43 did not co-localize with these granules. Under chronic, lower-dose arsenite, SGs again formed (with recruitment of TDP-43) but no differences were observed between control and patient cells. Notably, chronic stress led to cytoplasmic accumulation of phosphorylated TDP-43 that did not co-localize with TIAR-positive SGs, again without genotype-specific differences. Finally, Onda-Ohto et al. [36] quantified SG formation after H₂O₂ treatment: MNs from a control line edited with the *TARDBP* A382T mutation produced significantly more SGs than the isogenic control line. Although TDP-43 localization within these SGs was not assessed, the increased SG burden correlated with elevated H₂O₂-induced mortality in the mutant MNs.

Overall, spontaneous SG formation at baseline is inconsistent across *TARDBP*-mutant MN studies, with some reporting no differences and others showing modest increases. Under defined stress paradigms, SG induction kinetics are often similar between genotypes, but *TARDBP* mutants can exhibit context-dependent specific altered dynamics. These findings support SG dysregulation as a context-dependent phenotype that emerges under specific stress and recovery conditions rather than uniformly at rest. Consequently, SG-related endpoints are most informative when standardized stressors, recovery windows, and quantitative readouts are employed.

### Cellular mechanisms of ALS pathophysiology revealed using this model

#### Highlighted pathways from OMICS studies on ALS *TARDBP* motor neurons

RNA sequencing has been widely employed in *TARDBP*-mutated iPSC-derived MNs to identify dysregulated pathways (Table 9). A common finding across all studies is that MNs carrying *TARDBP* mutations, whether introduced via gene editing or derived directly from patient cells, exhibit extensive transcriptional changes. Compared to other ALS-related mutations, *TARDBP* mutations consistently result in a significantly higher number of differentially expressed genes (DEGs), underscoring the crucial role of TDP-43 in general RNA metabolism. Notably, RNA metabolism emerges as the most commonly dysregulated pathway across all studies included in this review. However, there is a divergence in findings regarding whether RNA metabolism is predominantly upregulated or downregulated. Studies such as Egawa et al. [46], Dash et al. [71], and Schweingruber et al. [82] report an upregulation of RNA metabolism, whereas Ziff et al. [43], Catanese et al. [78], and Valdebenito-Maturana et al. [73] describe its downregulation. These discrepancies may arise from differences in enrichment analysis methods and the specific aspects of RNA metabolism considered in each study.

**Table 9:** Omics results obtained from *TARDBP* mutant motor neurons

Author	Year	Mutations	Coating	Purity	Day of post-mitotic MN specification		Days at analysis	RNAseq	Upregulated	Downregulated
Egawa et al. [46]	2012	G298S (M55) M337V (M64) Q343R (F62)	PLL, Laminin Fibronectin	MAP2+ : 60% GFAP+ : 10% SMI-32+ : 30%		~ 29	~ 60	Bulk RNAseq	RNA metabolism	Intermediate filament organization Steroid metabolism
Mitsuzawa et al. [69]	2021	M337V (F62) Q343R (M55) N345K (M63) G376D (M36)	/	No quantification		19	33–40	Bulk RNA-seq of soma and axonal fractions separately	Chloride transmembrane transport Positive regulation of MAPK cascade Neuron fate commitment	Cell motility Postsynaptic membrane potential Positive regulation of axon extension Actin filament bundle assembly
Smith et al. [70]	2021	Q331K M337V	PLO Laminin	60%: MAP2 30%: Islet+		25–30	40–90	Bulk RNA-seq	Angiogenic process Hypoxic processes	Nervous system development
Dash et al. [71]	2022	G294V (M46) S393L (F85)	/	80–90% TuJ1+ 80–90% MAP2+ 70–75% SMI32+		20	30	Bulk RNA-seq	RNA metabolism DNA damage	/
									Mitochondrial dysfunction Oxidative stress DNA repair Protein transport Apoptosis	
Valdebenito-Maturana et al. [73]	2022	M337V (/)	/	> 70% HB9+		28	35–42	Bulk RNA-seq	Cell adhesion Cell communication Locomotion Cell surface signaling	Spinal cord motor neuron differentiation Visual and sensory perception RNA metabolism
Varderidou-Minasian et al. [74] **(Pre-print)**	2022	I383T (M60)	PDL Laminin	No quantification		20	20–60	Mass spectrometry	Antigen processing Actin filament depolymerization Neurodegeneration	mRNA splicing Mitochondrial respiratory chain complex Endocytosis Spliceosome function
Ziff et al. [43]	2023	/	/	/		/		Integration of Smith et al. [70] + Dafinca et al. [63]	DNA damage Synapse signaling Neuron projection	Protein binding Microtubule cytoskeleton RNA metabolism
Catanese et al. [78]	2023	G298S (M64) N390D (M)	Matrigel	No quantification		21	28	Bulk RNA-seq	Mucin glycosylation Retinoic acid signaling NF-κB signaling Apoptosis P53 pathway	Protein ubiquitination Synapse processing pathways Amino acid metabolism RNA metabolism
								WGBS	Hypomethylation Beta-oxidation Phagocytosis IFN-γ and integrin signaling	Hypermethylation Ion channel transport Glutamate binding Synapse organization Neuronal systems
Aly et al. [75]	2023	G298S (M64) N390D (M)	Matrigel	No quantification		21	42	Mass spectrometry of synaptic subcompartments	/	Spliceosome Metabolic pathways IMP catabolic processes Purine metabolic processes Cellular localization mRNA surveillance pathway
Schweingruber et al. [82]	2025	M337V (ed.)	PLO Laminin	> 95%: neurons 70%: MNs		14	21–28	Single-cell RNAseq	RNA metabolism Neuron projection organization Dendritic spine development	Energy metabolism (oxidative phosphorylation and aerobic respiration)
Lépine et al. [83]	2025	G348C (ed.) A382T (ed.)	PLO + Laminin	75%: HB9+ 60%: Islet+		21	49	Bulk RNA-seq	Ion binding DNA binding and transcription factor activity Cell adhesion	
Ma et al. [84]	2025	G298S (M64) A382T (F62)	PLO + Laminin	> 80%: Islet+ > 80%: HB9+		23	28–50	Bulk RNA-seq	Extracellular matrix organization Neuronal differentiation regulation Nonsense-mediated decay	

Summary of transcriptomic and proteomic studies performed in iPSC-MNs carrying *TARDBP* mutations. For each study, the mutation analyzed, coating conditions, neuronal purity, timing of post-mitotic MN specification, day of analysis, and omics approach used are indicated. The main upregulated and downregulated biological pathways identified in each dataset are summarized

Mutation notation. Mutations are indicated using protein-level nomenclature (e.g., M337V). When available, patient sex and age at biopsy are indicated in parentheses: M = male, F = female, and the number corresponds to age (years) at biopsy (e.g., M56). “ed.” indicates an isogenic gene-edited iPSC line (typically control iPSCs engineered to carry the indicated mutation)

“/” indicates that the information was not reported in the original study

Abbreviations: RNA-seq, RNA sequencing; WGBS, whole-genome bisulfite sequencing

A striking observation is that *TARDBP* mutations tend to drive more gene upregulation than downregulation, suggesting a strong transcriptional activation component associated with TDP-43 pathology. Egawa et al. [46] conducted the first transcriptomic analysis of *TARDBP*-mutant MNs and found a profound upregulation in RNA metabolism, including RNA binding, processing, and transcription initiation. This study also highlighted a significant downregulation in intermediate filament organization and steroid metabolism. The most notable finding was the imbalance in gene ontology (GO) terms, where approximately 430 terms were enriched in mutant MNs compared to only four downregulated terms, emphasizing the large-scale transcriptional alterations caused by *TARDBP* mutations.

Dash et al. [71] expanded on these findings by demonstrating that up to one-third of the transcriptome was dysregulated in patient-derived MNs. Their pathway enrichment analysis reinforced the strong upregulation of RNA metabolism, particularly spliceosome-related and transcriptional machinery genes. Additionally, they uncovered significant perturbations in mitochondrial function, oxidative stress response, DNA damage repair, protein transport, and apoptosis, which are key cellular processes implicated in ALS pathogenesis. Schweingruber et al. [82] used single-cell RNA sequencing in iPSCs edited to carry the M337V *TARDBP* mutation and found that MNs showed profound transcriptional differences compared to controls, with over 2200 DEGs. Interestingly, nearly all of these DEGs were unique to MNs and not found in interneurons, indicating a highly specific response in MNs. They identified 423 altered biological processes, with pathways such as cholesterol biosynthesis, ribonucleotide complex biogenesis, ncRNA export, and cellular amide metabolism regulation showing significant enrichment. Shared dysregulated processes with *FUS*-mutated lines included RNA metabolism upregulation and downregulation of energy metabolism, particularly oxidative phosphorylation and aerobic respiration. Furthermore, Lépine et al. [83] performed whole-transcriptome RNA sequencing of iPSC-derived MNs carrying *TARDBP* knock-in mutations (A382T and G348C) and demonstrated that these disease-associated variants induce widespread transcriptomic remodeling. In addition to significant alterations in mRNA expression, the authors identified marked changes in microRNA profiles, notably affecting the 14q32 microRNA cluster, indicating that *TARDBP* mutations impact both transcriptional and post-transcriptional regulatory layers. The study identified the NEDD8-activating enzyme inhibitor MLN4924 as a compound predicted to reverse the mutant transcriptional profile. Another recent study performed an integrated temporal transcriptomic analysis of iPSC-derived MNs carrying multiple ALS-associated mutations, including *TARDBP*, *C9orf72*, *FUS*, and *SOD1*, allowing direct comparison of mutation-specific and shared molecular signatures across disease subtypes. In *TARDBP* mutant MNs, the authors identified prominent alterations in pathways related to extracellular matrix organization**,** regulation of neuronal differentiation**,** and nonsense-mediated mRNA decay, indicating that TDP-43 dysfunction affects both neuronal identity programs and RNA surveillance mechanisms [84].

On the other hand, Ziff et al. [43] performed an integrative transcriptomic analysis by pooling publicly available data from multiple studies, incorporating data from both familial and sporadic ALS cases. In this large-scale meta-analysis, they found that synapse signaling and neuron projection processes were upregulated in *TARDBP* mutant MNs, whereas protein binding, microtubule cytoskeleton organization, and RNA metabolism pathways were downregulated. Importantly, they identified DNA damage as a uniquely shared dysregulated pathway across ALS iPSC models and post-mortem tissues, particularly in *TARDBP* mutants. Their study also revealed that *TARDBP* mutations resulted in over 3500 DEGs, far exceeding the numbers observed in other ALS-linked mutations such as *FUS*, *C9orf72*, and *SOD1*.

Catanese et al. [78] employed a multi-omics approach, integrating transcriptomics and epigenomics. They reported that at the transcriptional level, TDP-43 mutant MNs exhibited downregulation in RNA and amino acid metabolism, whereas pathways such as mucin glycosylation, retinoic acid signaling, and NF-κB signaling were upregulated. At the epigenetic level, they detected hypermethylation in promoters associated with ion channel transport, glutamate binding, synapse organization, and neuronal function. On the other hand, they detected hypomethylation in promoters related to beta-oxidation, phagocytosis, and interferon-gamma and integrin signaling. Their gene-centric validation using a line-by-line approach confirmed these findings, reinforcing the robustness of their multi-omics analysis.

Beyond transcriptomics, proteomic studies have provided complementary insights into the *TARDBP*-associated dysfunction. Varderidou-Minasian et al. [74] identified 107 upregulated and 57 downregulated proteins in *TARDBP* mutant MNs. The most highly dysregulated proteins are involved in cytoskeletal transport and synaptic vesicle recycling. GO enrichment analysis revealed an upregulation of antigen processing and actin filament depolymerization, while mRNA splicing and mitochondrial respiratory chain complex assembly were downregulated. KEGG pathway analysis highlighted neurodegeneration as an upregulated pathway, with endocytosis and spliceosome function being significantly downregulated. Aly et al. [75] further explored proteomic alterations at the synapses in *TARDBP-*mutant MNs. They found only a small subset of upregulated proteins, insufficient for pathway enrichment analysis. However, among the downregulated proteins, they identified strong enrichment for metabolic and spliceosomal pathways. When *TARDBP* patient lines were pooled with other ALS-linked mutations, mitochondrial transport and neurotransmitter secretion were consistently downregulated across all ALS subtypes. Additionally, their phospho-proteomics analysis revealed that while few phosphorylation sites were upregulated in *TARDBP* mutants, downregulated phosphosites showed enrichment in pathways related to cellular localization and mRNA surveillance.

In conclusion, *TARDBP* mutations profoundly impact the gene and protein expression in MNs, with RNA metabolism and synaptic dysfunction emerging as key dysregulated pathways. While transcriptomic analyses highlight widespread upregulation of genes, proteomic studies reinforce the importance of cytoskeletal, metabolic, and synaptic alterations in TDP-43-ALS pathology. The discrepancies observed between studies regarding RNA metabolism underscore the need for standardized analysis methodologies. Future efforts should focus on integrating transcriptomic and proteomic datasets to establish a unified understanding of TDP-43-driven neurodegeneration and uncover potential therapeutic targets.

#### TDP-43 splicing activity and cryptic exon expression in* TARDBP* mutant iPSC-MNs

As highlighted by the OMICS studies above, RNA metabolism is broadly perturbed in *TARDBP* mutant iPSC-MNs. This is consistent with the well-established role of TDP-43 in maintaining faithful gene expression by repressing the splicing of non-conserved cryptic exons [107], including in *UNC13A* and *STMN2* [108, 109]. A key question is therefore whether *TARDBP* mutations in iPSC-MN models are sufficient to recapitulate this splicing dysregulation, particularly the inclusion of cryptic exons, unannotated sequences that, when incorporated into transcripts, frequently introduce premature stop codons and trigger nonsense-mediated decay (NMD). Evidence from iPSC-MN models suggests that heterozygous *TARDBP* mutations produce only a partial and context-dependent recapitulation of this signature.

Klim et al. [59] reported that, while TDP-43 depletion robustly induced *STMN2* cryptic exon inclusion, *TARDBP* mutant MNs showed only modest downregulation of TDP-43 targets (including *STMN2*, *PFKP*, and *ELAVL3*), consistent with the preserved nuclear TDP-43 localization. Smith et al. [70] similarly found low but detectable *STMN2* cryptic exon inclusion in one *TARDBP*^Q331K^ line, but no inclusion in *TARDBP*^M337V^ or a second *TARDBP*^Q331K^ line, a discrepancy attributed to the absence of cytoplasmic TDP-43 mislocalization in the latter, highlighting the influence of genetic background.

Most directly, Held et al. [37] demonstrated in isogenic *TARDBP*^G298S/+^ MNs that, although 34 of 61 TDP-43-dependent splicing targets were downregulated, neither *STMN2* nor *UNC13A* cryptic exon inclusion was detectable by bulk RNA-seq at baseline; only pharmacological NMD inhibition (cycloheximide) allowed their accumulation. Consistent with this, Onda-Ohto et al. [36] found no significant changes in the splicing of classical TDP-43 targets *PDP1* and *BCL2L* in *TARDBP* mutant iPSC-MNs even under mild oxidative stress (low-dose H₂O₂), suggesting that TDP-43 splicing activity remains largely intact unless secondary stressors of greater magnitude are applied.

Beyond these canonical TDP-43 splicing targets, broader transcriptomic analyses revealed additional complexity. In the meta-analysis of 429 iPSC-MNs, Ziff et al. [43] identified *TARDBP* mutant lines as exhibiting the greatest number of alternative splicing changes of any ALS subgroup (1435 significant events), alongside downregulation of RNA splicing genes; yet none of the seven cryptic exons detected across the pan-ALS cohort overlapped with established TDP-43 depletion targets. Using eCLIP in sALS and fALS iPSC-MNs, Krach et al. [72] further showed that widespread alternative splicing dysregulation is driven substantially by loss-of-function of RNA-binding proteins such as NOVA1, NOVA2, and RBFOX2, rather than TDP-43 directly, whose eCLIP-binding sites are less enriched at differentially spliced loci.

Taken together, these findings indicate that heterozygous *TARDBP* mutations are insufficient to fully recapitulate TDP-43 loss-of-function splicing dysregulation at baseline, and that the cryptic exon signature characteristic of end-stage ALS tissue likely requires the cytoplasmic TDP-43 aggregation, NMD impairment, and chronic cellular stress that iPSC models do not yet replicate.

#### Mitochondria and metabolism alterations

Mitochondrial dysfunction has already been described as playing a role in motor neuron death in ALS [110]. Associated with TDP-43 specifically, abnormal mitochondrial trafficking, clustering and vacuolization have been observed in transgenic animals expressing mutant TDP-43 [111, 112], as well as in fibroblasts from ALS patients carrying *TARDBP* mutations [113–115]. In this context, several studies have focused on characterizing mitochondria shape, motility, function, expression and activity in *TARDBP* mutant MNs (Table 10).

**Table 10:** Mitochondrial alterations in *TARDBP* mutant motor neurons

Author	Year	Mutations	Parameter		Phenotype	Method
Kreiter et al. [55]	2018	G294V (M46) S393L (F87)	Size and shape of mitochondria	Altered	Altered	Mitotracker
			Motility	Altered	Decreased	Mitotracker
Fujimori et al. [57]	2018	M337V (M55) Q343R (F62)	ROS	Altered	Increased	CellROX
			Mitochondrial activity	Altered	Decreased	MTT assay
			Mitophagy	Altered	Decreased	
Sun et al. [58]	2018	G298S (M64)	Mitochondrial density in neurites	Altered	Decreased	Mitotracker
			Mitochondrial accumulation	Altered	Increased	IF
Yu et al. [65]	2020	G298S (M43) M337V (F62) A382T (M50)	mtDNA in cytoplasm	Altered	Increased	
			ROS	Altered	Increased	MitoSOX
			Membrane potential	Altered	Decreased	FACS with TMRM
Fazal et al. [68]	2021	G287S (F/) N290S (M/) A382T (F/)	Motility	Altered	Decreased	Mitotracker
			Mitochondrial density in neurites	**Unaltered**		Mitotracker
Smith et al. [70]	2021	Q331K (ed.) M337V (ed.)	Size and shape of mitochondria	Altered	Altered	Electron microscopy
			ROS	Altered	Increased	IF
			OCR	Altered	Decreased	Seahorse
Dafinca et al. [81]	2024	M337V (M57) I383T (M60)	OCR	Altered	Decreased	Seahorse
			Size of mitochondria	Altered	Decreased	Electron microscopy
			Retrograde mitochondrial and endosomal transport	Altered	Slower	Mitotracker
			Mitochondrial fusion	**Unaltered**	/	WB
Schweingruber et al. [82]	2025	M337V (ed.)	OCR	Altered	Decreased	Seahorse
			Motility	Altered	Decreased	TMRM dye
			Expression of mitochondrial genes	Altered	Downregulated	Transcriptomics

Summary of studies investigating mitochondrial phenotypes in iPSC-MNs carrying *TARDBP* mutations. The table lists the mitochondrial parameter assessed, the reported phenotype compared with control MNs, and the experimental method used

Mutation notation. Mutations are indicated using protein-level nomenclature (e.g., M337V). When available, patient sex and age at biopsy are indicated in parentheses: M = male, F = female, and the number corresponds to age (years) at biopsy (e.g., M56). “ed.” indicates an isogenic gene-edited iPSC line (typically control iPSCs engineered to carry the indicated mutation)

“/” indicates that the information was not reported in the original study

Abbreviations: ROS, reactive oxygen species; mtDNA, mitochondrial DNA; OCR, oxygen consumption rate; IF, immunofluorescence; WB, Western blot; FACS, fluorescence-activated cell sorting; TMRM, tetramethylrhodamine methyl ester

Mitochondrial size and shape are altered in *TARDBP* mutant MNs. Kreiter et al. [55] reported increased mitochondrial size and elongation, most evident in distal neurites of aged *TARDBP* MNs using MitoTracker. Consistently, Smith et al. [70] observed larger, elongated mitochondria with disrupted cristae by electron microscopy. By contrast, Dafinca et al. [81] also using electron microscopy, found smaller mitochondria (reduced average area) in MNs from a patient harbouring the M337V *TARDBP* mutation.

Mitochondrial motility along neurites is likewise perturbed. Fazal et al. [68] detected reduced mitochondrial motility in *TARDBP* MNs compared with controls, despite unchanged neuritic mitochondrial density. Sun et al. [58] reported decreased mitochondrial density in proximal neurites. Kreiter et al. [55] also observed reduced motility, which was more pronounced in aged MNs. In Dafinca et al. [81], motility defects emerged only under glutamate exposure and not at baseline.

Regarding function, extracellular flux (Seahorse) assays revealed impaired respiration. Smith et al. [70] reported decreased basal oxygen consumption rate (OCR) in *TARDBP* MNs, noting that very pure MN cultures did not survive the assay. Schweingruber et al. [82] likewise showed reduced basal and maximal respiration with preserved glycolysis. Dafinca et al. [81] confirmed decreased basal respiration in two *TARDBP* lines, while the maximal respiration and the spare respiratory capacity were unchanged. Reduced basal OCR has also been observed in other sALS and fALS MNs [116].

Transcriptomic data support mitochondrial involvement. Schweingruber et al. [82] reported downregulation of nearly half of mitochondrial genes in *TARDBP* MNs, potentially explaining the reduced respiration observed in that study (Table 9). Conversely, Dafinca et al. [81] found upregulation of ATP metabolism and electron transport chain constituents (ATPB and COX5A), validated by western blotting; expression of other mitochondrial genes was not reported. Additional mitochondrial parameters are altered in *TARDBP* MNs. Fujimori et al. [57] observed decreased mitochondrial activity by MTT assays (also seen in *FUS* MNs). Yu et al. [65] reported a reduced mitochondrial membrane potential. Increased basal reactive oxygen species (ROS) were detected in several studies [57, 65, 70].

In conclusion, several studies have reported altered morphology, density, motility and activity of mitochondria in MNs derived from *TARDBP* ALS patients. One hypothesis that could explain mitochondrial dysfunction in TDP-43-ALS is the abnormal accumulation of TDP-43 in the mitochondria. Indeed, Dafinca et al. [81] actually observed significantly increased co-localization of TDP-43 with mitochondrial proteins in *TARDBP* MNs compared to controls. Existing literature further discusses this hypothesis [65, 117]. However, in a co-immunoprecipitation experiment, mitochondrial proteins were not detected as partners of TDP-43 [81]. Mitochondrial alterations in *TARDBP* MNs are broad and not debated, however, further studies are needed to clarify if these alterations are a cause or a consequence of TDP-43-ALS pathogenesis.

### *TARDBP* mutant MNs as a drug screening tool: identified drugs and relevance of the model

In only the second study ever published on iPSC-derived MNs from *TARDBP* patients [46], researchers used this model as a drug screening tool, highlighting its potential for accelerating drug discovery. Below we list all the drug screening approaches that have included iPSC-derived MNs from *TARDBP* ALS patients specifically and detail their results (Table 11).

**Table 11:** Drugs tested in *TARDBP* mutant iPSC-derived motor neurons

Author	Year	Mutations	Number of drugs	Drug	Role	Effect	
Egawa et al. [46]	2012	G298S (M55) M337V (M64) Q343R (F62)	4	Trichostatin A	Histone deacetyltransferase inhibitor	No effect	
				Spliceostatin A	Spliceosomal factor inhibitor	No effect	
				Anacardic acid	Histone acetyltransferase inhibitor	Rescued arsenite-induced cell death in *TADRBP* MNs Decreased TDP-43 mRNA levels and TDP-43 levels in the insoluble fraction Increased neurite length Rescued transcriptome alterations	
				Garcinol	Histone acetyltransferase inhibitor	No effect	
Fujimori et al. [57]	2018	M337V (M55) Q343R (F62)	95	Ropinirole	Anti-Parkinson drug	Rescued neurite length, CC3 staining, pTDP-43 inclusions, LDH leakage	
Fang et al. [60]	2019	G298S (M) N352S (F59, M45, M53)	10	Cycloheximide		Inhibition of SG formation, no effect on TDP43 cytoplasmic puncta	
				Mitoxantrone		Inhibition of SG formation, decreased TDP-43 cytoplasmic puncta	
				Pyrvinium		Inhibition of SG formation, decreased TDP-43 cytoplasmic puncta	
				Digitoxin		No effect on TDP43 cytoplasmic puncta	
Yu et al. [65]	2020	G298S (M43) M337V (F62) A382T (M50)	1	H-151	STING inhibitor	Prevented TNF increase, prevents excess MN death	
Choi et al. [66]	2020	A90V (/) M337V (/) Q343R (/)	1	I-2	Inhibition of PP1 by inhibitor 2	Inhibited mitochondrial fragmentation Supressed neuronal toxicity	
Fazal et al. [68]	2021	G287S (F/) N290S (M/) A382T (F/)	1	Tubastatin A	HDAC6 inhibitor	Reduced levels of insoluble TDP-43 Reduction of the levels of CTF-35 and CTF-25 to normal Rescued cytoplasmic localization of TDP-43 Restored motility of axonal mitochondria	
Watts et al. [80]	2024	G298S (M43)	7	Riluzole	Approved ALS drug	No effect on ER-stress response	
				Edaravone	Approved ALS drug	No effect on ER-stress response	
				Kenpaullone	MN protective agent	Protected against ER stress-induced death and neurite degeneration	
				Mi-29	MAP4K4 inhibitor 29	Protected against ER stress-induced death and neurite degeneration	
				SP600125	Inhibitor of JNK	Protected against ER stress-induced death and neurite degeneration*	
				Enzastaurin	Inhibitor of PKC	Protected against ER stress-induced death and neurite degeneration*	
				GDC-0879	Inhibitor of BRAF	Protected against ER stress-induced death and neurite degeneration*	

Summary of pharmacological compounds evaluated in iPSC-derived motor neurons (MNs) carrying *TARDBP* mutations. For each study, the mutation analyzed, number of compounds screened, the drug tested, and its reported effects on TARDBP-related phenotypes are indicated. Outcomes include effects on neuronal survival, neurite morphology, TDP-43 pathology, stress granule formation, mitochondrial function, and stress-induced toxicity. *, compounds SP600125, Enzastaurin and GDC-0879 rescued ER stress-induced cell death in control neurons but were not tested in *TARDBP-*mutant patient cells

Mutation notation. Mutations are indicated using protein-level nomenclature (e.g., M337V). When available, patient sex and age at biopsy are indicated in parentheses: M = male, F = female, and the number corresponds to age (years) at biopsy (e.g., M56). “ed.” indicates an isogenic gene-edited iPSC line (typically control iPSCs engineered to carry the indicated mutation)

“/” indicates that the information was not reported in the original study

Abbreviations: SG, stress granule; CC3, cleaved caspase-3; LDH, lactate dehydrogenase; TNF, tumor necrosis factor; HDAC, histone deacetylase; STING, stimulator of interferon genes; PP1, protein phosphatase 1; JNK, c-Jun N-terminal kinase; PKC, protein kinase C; BRAF, B-Raf proto-oncogene serine/threonine kinase

In Egawa et al. [46], drug selection was guided by transcriptomics, which identified transcription and RNA-splicing as top altered pathways. Four transcription modulators were tested. Anacardic acid rescued the sodium-arsenite-induced cell death in patient lines and, at baseline, decreased the abnormal insoluble TDP-43, increased neurite length, and corrected defects in RNA metabolism and intermediate filament pathways (but not steroid biosynthesis).

Fujimori et al. [57] conducted the first high-throughput screen in ALS iPSC-derived MNs, testing 1232 approved drugs across sporadic and familial lines, including *TARDBP* mutants. A multiphenotypic readout assessed neurite length, cleaved-caspase-3 staining, phosphorylated TDP-43 inclusions, and LDH leakage. After a primary screen in *FUS* MNs, 95 candidates were re-tested in *TARDBP* MNs; nine compounds rescued phenotypes in both. Ropinirole (a dopamine D2 receptor agonist) was prioritized based on blood–brain barrier permeability, side-effect profile, and dose–response. A phase 1/2a trial in 20 sALS patients showed no benefit during the double-blind phase [118]; exploratory findings from the open-label extension suggested possible slowing of progression, warranting larger studies. Ropinirole has not been approved for ALS.

Fang et al. [60] used high-content screening to identify modulators of SG formation and dissociation. SG dynamics is considered a relevant therapeutic target in ALS because persistent SGs can promote the accumulation and aggregation of RNA-binding proteins such as TDP-43 as discussed earlier. Approximately 5000 compounds were screened in transgenic proliferative lines; 10 hits that inhibited SG formation were validated in iPSC-derived MNs. The SG inhibitors cycloheximide, mitotraxone, and pyrvinium suppressed SG formation in control and patient MNs. However, only mitotraxone and pyrvinium produced lasting reductions in cytoplasmic TDP-43 puncta in *TARDBP* MNs after stress washout.

In Watts et al. [80], researchers amplified latent vulnerabilities by inducing endoplasmic reticulum (ER) stress with thapsigargin and tunicamycin. *TARDBP* and *SOD1* MNs tended to show increased vulnerability under ER stress. This ER-stress response was then used as a screening readout to identify neuroprotective agents. Riluzole and edaravone did not rescue the ER-stress-induced apoptosis in control lines (no data were shown for *TARDBP* and *SOD1* lines). By contrast, kenpaullone improved the ER-stress-induced cell death and preserved neurite networks in both control and patient MNs. Inhibiting MAP4K4 (kenpaullone’s direct target) with Mi-29 produced similar rescue. Phospho-proteomics implicated JNK, PKC, and BRAF as kenpaullone-modulated nodes; selective inhibition of each was sufficient to rescue the ER-stress phenotype in control lines.

Beyond screening campaigns, several studies assessed single candidates grounded in specific disease mechanisms. Yu et al. [65] showed that STING inhibition with H-151 rescued abnormal cell death at day 26, linking mitochondrial TDP-43, cytosolic mtDNA release, and neuroinflammation. Choi et al. [66] reported that PP1 suppression with I-2 inhibited mitochondrial fragmentation and reduced neuronal toxicity (fewer pyknotic and caspase-3-positive cells), targeting Drp1 dysregulation. Fazal et al. [68] demonstrated that HDAC6 inhibition with Tubastatin A reduced cytoplasmic TDP-43 mislocalization and phosphorylation, normalized TDP-43 levels, and rescued mitochondrial transport defects.

Drug screening efforts on iPSC-derived MNs from *TARDBP* patients have not yet led to any of the tested drugs to be approved as a treatment for ALS. The iPSC-derived MNs from other fALS (*C9orf72* and *FUS*) and sALS patients have also been used for drug screening (reviewed in [119], with similar conclusion). Nevertheless, iPSC-modelling in ALS has led to 3 drugs being tested in patients in clinal trials [120]. The main challenge stems for the need of better markers for drug screening efforts. For example, the drug screening study on *TARDBP* MNs that led to the ropinirole clinical trial [57], focused on the rescue of the affected neurite length, basal mortality levels and phosphorylated TDP-43 accumulations in *TARDBP* patient lines. However, as discussed in detail in this review, these three parameters are still very much debated in the field, and were found to be not affected in *TARDBP* MNs in several studies. Given the ongoing uncertainty surrounding reliable biomarkers, an alternative approach would be to enhance certain phenotypic parameters that are consistently observed across patient lines. Indeed, the only parameter that is not debated in the *TARDBP* iPSC-derived MN field is their increased sensitivity and susceptibility. This was for example done in the study from Watts et al. (2024) [80] using ER stressors, but other pathways could be targeted. This approach could make for more reliable and robust drug screening approaches.

### Non-cell autonomous mechanisms of disease: What about other cell types?

In recent years, researchers have examined the contribution of non-MN cell types to the pathogenesis of ALS. This has led to the recognition that other cell types, such as glial cells, skeletal muscle or the immune system, may contribute to the disease. In this context, the ALS research field has tried to model how *TARDBP* mutations, among others, affect the phenotype of other cell types than MNs by differentiating patient iPSCs into the different targeted cell types. These studies are listed in Table 12.

**Table 12:** Differentiation of *TARDB*P mutant iPSCs into other cell types

Author	Year	Mutations	Cell type	Purity	Phenotype
Serio et al. [121]	2013	M337V (M56)	Astrocytes	> 90%: GFAP+ / S100β+	**Functional** **Non-toxic to MNs**
					Increased cytoplasmic TDP-43 *Higher risk of cell death*
Arredondo et al. [122]	2022	A90V (F75)	Astrocytes	91%: Cx43+ 93%: EAAT+ 78%: s100b+	*Increased levels of PolyP* *ACM toxic to MNs*
Rojas et al. [123]	2023	A90V (F75)	Astrocytes	> 95%: ALDH1L1+ > 80%: s100b+ > 95%: EAAT2+ > 90%: Cx43+ < 15%: GFAP+	**No TDP43 inclusions**
					*Increased cytoplasmic TDP-43* *Reactive state* *ACM toxic to MNs*
Ferraiuolo et al. [124]	2016	G298S (M63)	Oligodendrocytes	82%: O4+ 90%: GalC+	*Co-culture toxic to MNs* + + *ACM toxic to MNs* *Decreased lactate production* *SOD1 aggregates*
Barton et al. [125]	2021	G298S (M64) M337V (M56)	Oligodendrocytes	60–80%: OLIG2+ 40–60%: O4+	**Normal TARDBP mRNA levels** **Normal morphology** **Normal metabolism** **Normal myelination**
					*Increased cytoplasmic TDP-43*
Matsuo et al. [126]	2024	N345K (M63) G295S (ed.) K263E (ed.)	BMECs	/	Phenotypes only in N345K mutation: *Increased cytoplasmic TDP-43* *Increased permeability* *Increased immune cell adhesion molecules*
Lenzi et al. [127]	2016	A382T (M50)	Muscle	/	**Normal electrical properties**
					*Reduced Ach-evoked currents*
Lynch et al. [128]	2021	G298S (M43)	Muscle	/	*Dysregulated vesicle-mediated transport*
Osaki et al. [129]	2018	G298S (F55)	Motor unit	/	*Delayed neurite outgrowth* *Fewer thick nerve fibers* *Increased apoptosis and atrophy in the muscle* *Reduced contraction force of the muscle*
Melamed et al. [130]	2019	N352S (/) G298S (/) A382T (/) N390S (/)	Cortical neurons	/	*Increased cytoplasmic TDP-43* *Down-regulation of stathmin-2 mRNA levels*
Imaizumi et al. [131]	2022	K263E (ed.)	Cortical neurons	/	**Nuclear localization of TDP-43**
					*RNA process alterations* *Down-regulation of stathmin-2 mRNA levels*
Fang et al. [132]	2023	M337V (M64) Q343R (F62)	Cortical neurons	/	*Increased DNA damage levels* *Increased susceptibility to DNA damaging agents*

Summary of studies in which *TARDBP*-mutant iPSCs were differentiated into non-motor neuron cell types, including astrocytes, oligodendrocytes, brain microvascular endothelial cells (BMECs), muscle cells, motor units, and cortical neurons. For each study, the mutation analyzed, cell purity, and reported phenotypes compared with controls are indicated. **Italics** denote altered or disease-related phenotypes, whereas **Bold** indicate no detectable differences

Mutation notation. Mutations are indicated using protein-level nomenclature (e.g., M337V). When available, patient sex and age at biopsy are indicated in parentheses: M = male, F = female, and the number corresponds to age (years) at biopsy (e.g., M56). “ed.” indicates an isogenic gene-edited iPSC line (typically control iPSCs engineered to carry the indicated mutation)

“/” indicates that the information was not reported in the original study

Abbreviations: ACM, astrocyte-conditioned medium; GFAP, glial fibrillary acidic protein; S100β, S100 calcium-binding protein β; Cx43, connexin 43; EAAT2, excitatory amino acid transporter 2; ALDH1L1, aldehyde dehydrogenase 1 family member L1; O4, oligodendrocyte marker; OLIG2, oligodendrocyte transcription factor 2; GALC, galactocerebrosidase

#### Astrocytes

Astrocytes play a crucial role in the central nervous system (CNS), providing metabolic support, structural integrity, and neuroprotection to neurons. Research has shown that astrocytes contribute to the onset and progression of ALS in various models. However, most of the available in vivo and in vitro data on astrocyte involvement in ALS came from SOD1 models, while findings related to TDP-43-ALS remain controversial [133]. To better understand the role of astrocytes in ALS pathogenesis, researchers have generated iPSC-derived astrocytes carrying *TARDBP* mutations.

Serio et al. [121] differentiated iPSCs from an ALS patient carrying the M337V mutation into astrocytes and found no differences in differentiation efficiency between patient and control lines. These patient-derived astrocytes functioned similarly to controls, maintaining the ability to propagate calcium waves and promote synapse formation in co-culture with MNs. While they did not exhibit altered *TARDBP* mRNA expression, they showed increased levels of soluble TDP-43 protein, with nuclear TDP-43 levels remaining unchanged but cytoplasmic levels elevated. Importantly, the M337V astrocytes had a 2.5-fold higher risk of cell death compared to controls, and caspase inhibition only partially prevented this. Despite these changes, mutant astrocytes did not exert toxicity on MNs in co-culture.

Arredondo et al. [122] studied astrocytes derived from iPSCs carrying the A90V mutation and found elevated levels of polyphosphate (PolyP), a molecule hypothesized to be toxic in ALS. When MNs were exposed to conditioned media from these mutant astrocytes, neuronal death increased. However, this effect was reversed by treating the media with PolyP-degrading enzymes, suggesting a role for PolyP in ALS-related toxicity.

Rojas et al. [123] highlighted a critical issue in ALS research: most differentiation protocols produce immature and reactive astrocytes, making them less suitable for disease modelling. To address this, the study generated functional, mature astrocytes with low GFAP expression, a marker of astrocyte reactivity. These astrocytes showed similar differentiation patterns between patient and control lines. Immunocytochemistry revealed increased cytoplasmic localization of TDP-43 in patient-derived astrocytes, though no aggregates or inclusions were detected. Unlike controls*, TARDBP* mutant astrocytes displayed mild signs of an intrinsic reactive state. Furthermore, while the conditioned media from control astrocytes promoted synapse maturation in hippocampal neurons, the media from *TARDBP* astrocytes failed to do so. Additionally, conditioned media from mature *TARDBP* astrocytes was sufficient to induce motor neuron death in spinal cord cultures, though interneurons remained unaffected.

In conclusion, while some studies suggest that *TARDBP* mutant astrocytes do not directly harm MNs, others indicate their potential to release toxic factors like PolyP, impair synapse maturation, and promote motor neuron death. These findings further add to the already-existing controversy related to the involvement of astrocytes in TDP-43-ALS.

#### Oligodendrocytes

Oligodendrocytes are the myelinating cells in the CNS, making them important players in signal propagation along axons in the brain, among other roles. Importantly, demyelination is a process that has been observed and quantified in ALS patients, as well as in animal models of ALS [134]. How *TARDBP* mutations specifically affect iPSC-derived oligodendrocytes is summarized in this paragraph.

Ferraiuolo et al. [124] differentiated oligodendrocytes from a *TARDBP* G298S iPSC line and co-cultured them with HB9-GFP⁺ MNs, observing a 40%–60% reduction in MN viability compared to controls. Exposure of MN monocultures to conditioned medium from *TARDBP* oligodendrocytes also reduced the viability, though to a lesser extent. Mechanistically, *TARDBP* oligodendrocytes produce significantly less lactate than control lines after several weeks of maturation. Lactate supplementation to conditioned medium fully rescued MN viability, while in co-culture it only partially prevented MN loss, suggesting that additional non-cell-autonomous mechanisms contribute to the toxicity. Interestingly, *TARDBP* oligodendrocytes also display abnormal SOD1 protein aggregates, and *SOD1* knockdown restored both lactate production and MN survival. These results suggest SOD1 as a potential downstream therapeutic target in TDP-43-associated ALS.

Barton et al. [125] investigated oligodendrocytes derived from two *TARDBP* ALS patients and found evidence of cytoplasmic mislocalization of TDP-43 in the form of inclusions, while *TARDBP* mRNA levels remained unchanged. During normal oligodendrocyte maturation, AMPA receptors (AMPARs) transition from being Ca^2+^-permeable to Ca^2+^-impermeable. This transition occurred in control and M337V *TARDBP* oligodendrocytes but was absent in the G298S line. Notably, correcting the G298S mutation restored normal AMPAR transition. Despite this, G298S oligodendrocytes exhibited normal morphology, maturation, and metabolism as measured by Seahorse glycolysis assay. Furthermore, when assessed in an organoid model, *TARDBP* G298S oligodendrocytes did not show defects in myelination compared to controls.

To conclude, these studies highlight the complex effects of *TARDBP* mutations in oligodendrocytes. While one study suggests metabolic defects concerning lactate production, the other study did not observe these metabolic alterations on glucose production. One study shows that *TARDBP* oligodendrocytes lead to MN death, while the other shows that myelination properties are not affected. These studies examined only one or two patient lines at a time, making it challenging to draw broader conclusions about TDP-43-ALS. Future research on iPSC-derived oligodendrocytes should incorporate a larger number of patient lines to determine whether different *TARDBP* mutations lead to distinct phenotypes.

#### Brain microvascular endothelial cells (BMECs)

MNs are protected in the CNS from circulating toxins and pathogens, but also from unwanted immune cells by the blood–brain barrier (BBB). This barrier consists mainly of BMECs, characterized by continuous tight junctions, that regulate immune cell migration. Abnormal presence of immune cells in the CNS of post-mortem tissues from ALS patients suggests BBB alterations [135].

In this context, Matsuo et al. [126] differentiated iPSCs harbouring 3 *TARDBP* mutations into BMECs to assess cell-autonomous implications of the BBB in ALS pathogenesis. This study showed that the N345K heterozygous *TARDBP* mutation led to the loss of tight junctions and increased permeability of iPSC-derived BMECs to small molecules. Moreover, N345K *TARDBP* mutant BMECs had increased expression levels of adhesion molecules targeted to immune cells. All these functional alterations were accompanied by abnormal mislocalization of TDP-43 in the cytoplasm of these cells. Importantly, BMECs harbouring the 2 other K263E and G295S homozygous *TARDBP* mutations did not recapitulate any abnormalities.

#### Muscle and neuromuscular junction (NMJ)

ALS is characterized by MN degeneration, and consequently is also characterized by NMJ degeneration and skeletal muscle atrophy. It has been hypothesized that degeneration of the muscle or the NMJ could happen prior to MN death, and that the disease actually starts at the periphery and causes a dying-back process on the MNs [136]. iPSCs offer the opportunity to directly study the muscle or the NMJ of patients in vitro.

Only two studies have explored iPSC-derived skeletal muscle with *TARDBP* mutations. Lenzi et al. [127] differentiated skeletal muscle from an iPSC line carrying a homozygous A382T mutation and found that these muscle cells had normal passive electrical properties, including membrane capacitance and resting membrane potential. However, acetylcholine-evoked currents were reduced despite similar expression of muscle markers between patient and control lines. Lynch et al. [128] differentiated iPSCs derived from familial (G298A *TARDBP*, *C9orf72*, *SOD1*) and sporadic ALS lines into skeletal myocytes, and observed no differences in differentiation efficiency. RNA sequencing revealed only four commonly dysregulated genes across all ALS lines, all linked to vesicle-mediated transport. No further analysis was conducted on *TARDBP* mutant myocytes.

Studies on iPSC-derived skeletal muscle from *TARDBP* patients are limited and lack detailed functional analysis. Given the potential role of muscle dysfunction in ALS, further research is needed to better understand how *TARDBP* mutations impact muscle physiology and their contribution to disease progression.

Three-dimensional (3D) models of motor units provide a more physiologically relevant system to study ALS by integrating MNs with muscle fibers. Osaki et al. [129] developed a 3D motor unit model using iPSC-derived MNs from an sALS patient with the *TARDBP* G298S mutation. First, in 2D cultures, patient MNs had reduced Islet1^+^ cells, shorter neurites, increased *TARDBP* mRNA, and TDP-43 protein aggregation. When cultured in 3D MN-spheroids, patient MNs showed lower CHAT protein levels, even though the HB9^+^ cell percentages remained unchanged. When co-cultured with muscle fibers in a microfluidic device, *TARDBP* MNs exhibited delayed neurite outgrowth and formed fewer thick nerve fibers. The motor unit showed increased apoptosis, muscle atrophy, and reduced contraction force. Notably, treatment with rapamycin or rapamycin plus bosutinib improved both muscle atrophy and contraction strength.

#### Cortical neurons

ALS shares characteristics with another neurodegenerative disease, frontotemporal lobar degeneration (FTLD). This disease is characterized by progressive atrophy of the frontal and temporal lobes. This leads to behavioral and personality changes, making FTLD the second most common type of dementia after Alzheimer’s disease [137]. Importantly, TDP-43 is also a major component of the cytoplasmic inclusions found in around 50% of FTLD patients. In this context, iPSCs harbouring *TARDBP* mutations have also been differentiated into post-mitotic neurons to model alterations in a different type of neurons than MNs.

Melamed et al. [130] induced neurons directly from ALS patient fibroblasts harbouring various *TARDBP* mutations. In the patient fibroblasts, TDP-43 was almost exclusively nuclear, while in the induced neurons, TDP-43 was mislocalized in the cytoplasm of neurons, and this mislocalization was enhanced in neurons harbouring the N352S mutation (data not shown for the other mutations). Moreover, induced neurons from all these patients showed an important down-regulation of stathmin-2 mRNA levels, a protein involved in neurite outgrowth, whose splicing is known to be regulated by TDP-43.

Imaizumi et al. [131] only focused on the K263E *TARDBP* mutation identified in a FTLD patient, and differentiated the iPSC line into cortical neurons. Using transcriptomics, they identified impairments in important RNA processes, with stathmin-2 levels being significantly decreased in the K263E cortical neurons. Importantly, TDP-43 subcellular localization was not altered with a strong nuclear localization of the protein.

Lastly, Fang et al. [132] found that *TARDBP* mutated neurons had increased DNA damage levels, which could be explained by defects in DNA double-strand break repair. These neurons were also more sensitive to DNA damaging agents etoposide and 5-fluorouracil.

## Discussion and perspectives

Studies of iPSC-derived MNs harboring *TARDBP* mutations have yielded important mechanistic clues, yet our systematic review makes clear that the literature is marked by substantial discrepancies. While it is intuitive to attribute these differences to variation in differentiation protocols, neuronal purity, maturation state and coating, we could not establish consistent correlations between the reported phenotypes and these variables across studies, largely because the methodological frameworks and analysis choices differed and the necessary metadata were often incompletely reported. Instead, the most reliable driver of divergent outcomes was the analysis methodology itself. Certain quantification approaches systematically failed to detect particular phenotypes (for example, TDP-43 mislocalization under specific image-analysis pipelines) while others reported small effects. This indicates that sensitivity, thresholding, and region-of-interest selection can shape conclusions as much as the underlying biology.

From this, we learned that cross-study comparisons are fragile unless acquisition and analysis are standardized and transparently documented, and that conclusions based on non-automated, variably parameterized image or signal processing should be treated with caution. In this context, we advise that future studies should prioritize automation at every step: (1) defining acquisition settings and segmentation thresholds, (2) using automated analysis pipelines applied identically across lines, (3) incorporating isogenic controls and multiple independent mutant lines within the same study, which should undergo differentiation at the same time in same batches, (4) reporting neuron subtype composition and purity with agreed markers, and (5) sharing raw data and code so results can be re-analyzed. We believe that these measures, together with clear reporting of antibodies, imaging parameters, and criteria for regions of interest, will reduce the experimenter bias, enable meaningful meta-analysis, and help the field converge on truly reproducible phenotypes. In parallel, microfluidic culture systems represent a promising standardized platform to model axonal compartments and transport processes in a controlled and reproducible manner, which may improve the study of axonal pathology and facilitate cross-study comparability [138, 139].

Despite these challenges, based on this review, three phenotypes emerge with the greatest consistency: (1) heightened sensitivity to exogenous stressors, (2) mitochondrial structural/functional deficits, and (3) disturbances of RNA metabolism. Taken together, these convergent findings support the validity of mutant *TARDBP* iPSC-MNs for modelling TDP-43-related ALS. For studies requiring quantitative readouts (especially drug screening), these features should be prioritized as primary endpoints, whereas measures such as TDP-43 mislocalization and neurite morphology remain variable across studies and are not recommended as primary screening metrics. Crucially, these conclusions are specific to *TARDBP* iPSC-derived MN models and should not be generalized to other genetic forms (e.g., *C9orf72*, *SOD1*, *FUS*) or to sALS.

Electrophysiological studies have revealed a dynamic progression in motor neuron excitability, with some studies reporting initial hyperexcitability followed by progressive hypoexcitability and synaptic dysfunction. This aligns with in vivo findings from transgenic rodent models and human ALS patients, suggesting that intrinsic electrical dysfunction is a key component of ALS pathogenesis. However, the divergence in results across different iPSC studies underscores the necessity of refining experimental conditions to achieve more consistent results. Future research should aim to determine whether the observed excitability differences are dependent on the specific *TARDBP* mutation, neuronal maturation state, or experimental setup.

Another major finding in iPSC-derived *TARDBP* MNs is the widespread transcriptional and proteomic dysregulation, particularly affecting RNA metabolism, mitochondrial function, and cytoskeletal integrity. The observed upregulation of RNA processing genes and downregulation of oxidative phosphorylation pathways reinforce the hypothesis that *TARDBP* mutations disrupt cellular homeostasis on multiple levels. Despite some inconsistencies across studies, transcriptomic meta-analyses have identified shared pathways that may serve as potential therapeutic targets.

Drug screening efforts using iPSC-derived MNs have led to promising candidates, but no approved treatments have emerged from these studies. The recent clinical testing of ropinirole, identified through high-throughput screening, shows both the potential and the limitations of this approach. The lack of clear biomarkers for drug efficacy remains a major obstacle. Future studies should prioritize the identification of reliable functional readouts that can predict clinical outcomes, ensuring that drug candidates selected in vitro have a better chance to be effective in patients.

In conclusion, iPSC-derived MNs from *TARDBP* ALS patients have significantly advanced our understanding of ALS pathophysiology, despite variability in reported findings. Standardization of differentiation protocols, improved electrophysiological characterization, and integration of multi-omics data will be crucial for refining this model and enhancing its utility for therapeutic development. Additionally, expanding studies to include non-cell autonomous mechanisms by incorporating glial cells and neuromuscular junction models could provide a more comprehensive understanding of disease progression. Ultimately, continued advancements in iPSC technology and ALS modelling will be key to close the gap between in vitro findings and clinical applications.

## Data Availability

All data analyzed in this study are derived from published articles that are publicly available. No new datasets were generated or analyzed during the current study.
